# Incidence, Carriage and Case-Carrier Ratios for Meningococcal Meningitis in the African Meningitis Belt: A Systematic Review and Meta-Analysis

**DOI:** 10.1371/journal.pone.0116725

**Published:** 2015-02-06

**Authors:** Thibaut Koutangni, Halima Boubacar Maïnassara, Judith E. Mueller

**Affiliations:** 1 EHESP French School of Public Health, Sorbonne Paris Cité, Rennes, France; 2 Centre de Recherche Médicale et Sanitaire (CERMES), Niamey, Niger; 3 Institut Pasteur, Emerging Diseases Epidemiology Unit, Paris, France; University Hospital San Giovanni Battista di Torino, ITALY

## Abstract

**Background:**

To facilitate the interpretation of meningococcal meningitis epidemiology in the “African meningitis belt”, we aimed at obtaining serogroup-specific pooled estimates of incidence, carriage and case-carrier ratios for meningococcal meningitis in the African meningitis belt and describe their variations across the endemic, hyperendemic and epidemic context.

**Methods:**

We conducted a systematic review and meta-analysis of studies reporting serogroup-specific meningococcal meningitis monthly incidence and carriage in the same population and time period. Epidemiological contexts were defined as endemic (wet season, no epidemic), hyperendemic (dry season, no epidemic), and epidemic (dry season, epidemic).

**Findings:**

Eight studies reporting a total of eighty pairs of serogroup-specific meningococcal meningitis incidence and carriage estimates were included in this review. For serogroup A, changes associated with the transition from endemic to hyperendemic incidence and from hyperendemic to epidemic incidence were 15-fold and 120-fold respectively. Changes in carriage prevalence associated with both transitions were 1-fold and 30-fold respectively.  For serogroup W and X, the transition from endemic to hyperendemic incidence involved a 4-fold and 1•1-fold increase respectively. Increases in carriage prevalence for the later transition were 7-fold and 1•7-fold respectively. No data were available for the hyperendemic-epidemic transition for these serogroups. Our findings suggested that the regular seasonal variation in serogroup A meningococcal meningitis incidence between the rainy and the dry season could be mainly driven by seasonal change in the ratio of clinical cases to subclinical infections. In contrast appearance of epidemic incidences is related to a substantial increase in transmission and colonisation and to lesser extent with changes in the case-carrier ratio.

**Conclusion:**

Seasonal change in the rate of progression to disease given carriage together with variations in frequency of carriage transmission should be considered in models attempting to capture the epidemiology of meningococcal meningitis and mainly to predict meningitis epidemics in the African meningitis belt.

## Introduction

The epidemiology of bacterial meningitis in the African meningitis belt is characterized by regular hyperendemicity during one single dry season (approximately November-May), which alternates with endemic incidence during the rainy season (June-October). [1, 2] Epidemics of meningococcal meningitis occur on the community level irregularly, but always limited to the second half of the dry season. In cycles of 7–10 years, epidemics form waves that span larger regions and consecutive dry seasons. Until the introduction of a meningococcal serogroup A conjugate vaccine (MenAfriVac) in the meningitis belt from 2010 on, these epidemics were mostly due to serogroup A *Neisseria meningitidis* (NmA), but since then, no NmA epidemics have occurred. However, since 2000, serogroups W (NmW) and X (NmX) have repeatedly caused epidemics, sometimes with local incidence rates comparable to NmA epidemics. [3] The factors leading to epidemics remain hypothetic [4], but their identification would help to better predict epidemics and designing control strategies, including vaccination.

Several hypotheses exist as to why seasonality and seasonal epidemics occur [5–8], but apart from modelling studies of meteorological information and some opportunistic studies during outbreaks, no hypothesis-driven research has occurred. In a conceptual model for meningococcal epidemics in the meningitis belt, Mueller & Gessner [4] suggested that the transitions from endemicity (during the wet season) to seasonal hyperendemicity and sporadic epidemics (during the dry season) are two distinct phenomena caused by different mechanisms. These mechanisms would include increased risk of invasion given pharyngeal colonisation during the dry season, and surges in colonisation leading to epidemics.

Building on this model, we aimed at exploring how colonisation and susceptibility to meningitis given colonisation change over seasons and epidemics. Dynamics of colonisation can be estimated in carriage studies. The case-carrier ratio (CCR) is an ecological proxy for the risk of meningitis given colonisation and can be estimated by dividing meningitis incidence by concurrent carriage prevalence. We therefore conducted a systematic review with meta-analysis to provide best evidence on how serogroup-specific incidence, carriage and case-carrier ratio vary according to epidemiological context (endemicity, hyperendemicity and epidemic) in the African meningitis belt.

## Methods

This review was conducted based on an elaborated systematic review and meta-analysis protocol (S1 Text). Reporting is done according to the PRISMA 2009 checklist (S1 PRISMA Checklist). We aimed at including studies that (1) reported serogroup-specific meningococcal carriage and laboratory-confirmed meningococcal meningitis cases over the same time period in the same population; (2) were conducted in populations within the African meningitis belt; (3) included a representative sample of the general population for carriage evaluation (at least cluster sampling free of coverage bias) and enrolled suspected meningitis cases in exhaustive way; (4) were conducted from 1969 onward. Studies targeting children and/or young adults attending schools were also eligible provided that school attendance was common. We included only studies conducted after 1969, when the distinction between *N. meningitidis* and *N. lactamica* was possible. [9] We searched MEDLINE, Academic Search Complete via EBSCOhost and the African Index Medicus for medical subject headings and text words representing the concepts meningococcal meningitis, colonisation and African meningitis belt countries (S2 Text). Databases searches were initially performed in February 2012 and last updated in December 2013. Our selection criteria included publications in English and French languages. We hand searched references lists of included papers, relevant reviews and contacted relevant research groups to identify unpublished data. After a first screening based on titles and abstracts of retrieved records by one reviewer, two reviewers conducted full text screening and data extraction. Study and participants’ characteristics, as well as relevant meningococcal serogroup-specific data were extracted from eligible studies by one reviewer (Table 1, S1 Table). We used Graph Extract v2.5 (QuadTech Associates) for data extraction from graphs in two studies. [10, 11]

**Table 1:** Summary characteristics of included studies reporting meningococcal serogroup-specific incidence and carriage prevalence of the same population and time period.

**First author.Year [Reference]**	**Settings**	**Age range (years)**	**Sampling time point/ Follow-up**	**Study participants**	**Vaccination status of study population (date of vaccine campaign)¶**	**Epidemiological context of study / Season**
Boisier et al. 2006 [25]			May 2003			Hyperendemic / post-epidemic (first rains mid-May, humidity <40% until end of May)
	Djinguinis, Azao, Fardak and Dallé villages (Tahoua region, Niger)	2–65		Residents of villages referring to Illela health centre and having registered at least one NmW case during March and April 2003 in the district of Illela.	No	
			February 2004			Hyperendemic / Dry
Hamidou et al. 2006 [26]			February 2003			Hyperendemic / Dry
	Primary schools in Niamey (Niger)	7–16	March 2003	Primary schools children in Niamey	Yes (2001/2002)	Hyperendemic / Dry
			May 2003			Hyperendemic / Dry (first rains mid-may humidity <40% until end of May)
Hassan-King et al. 1987 [11]	Farafeni (Gambia)	2–20	January to April 1983	Residents living in two villages in the centre of the Farafeni study area.	No	Serogroup A epidemic / Dry
Leimkugel et al. 2007 [10]	Kessena Nankana district (Ghana)		April from1998 to 2005			Endemic / Wet
		< 5–50+		Inhabitants of Kessena Nankana district	Yes (1997/2005 yearly campaigns)	
			November from1998 to 2005			Hyperendemic / Dry
Mueller et al. 2011 [23]			March 2006	Residents of Kofila and Konkourouna	No	Serogroup A epidemic / Dry
	Lena, Kofila, and Konkourouna villages (Burkina Faso)	1–39				
			March 2006	Residents of Lena	Yes (March 12–15, 2006)	Serogroup A epidemic / Dry
Mueller et al. 2006 [22]	Urban Bobo-Dioulasso (Burkina Faso)	4–29	February, March, and April 2003	Residents of the urban area of sanitary districts Secteur 15 and Secteur 22 as of Feb-June 2003 (urban Bobo-Dioulasso)	Yes (2002)	Hyperendemic / Dry
Sié et al. 2008 [24]	Nouna district (Burkina- Faso)	not reported	April 2006	Resident of the Nouna Demographic Surveillance System Area	No	Hyperendemic / Dry
Trotter et al. 2013 [21]	Urban Bobo-Dioulasso (Burkina Faso)	0–59	February to March 2008	Residents of the urban area of Bobo-Dioulasso	No	Hyperendemic / Dry

^¶^ Yes, if the study population have been vaccinated within 2 weeks to 3 years prior to the onset of carriage and surveillance studies, using a vaccine against one or several meningococcal serogroups. All campaigns were conducted using serogroup A/C meningococcal polysaccharide vaccines.

**Figure 1:**
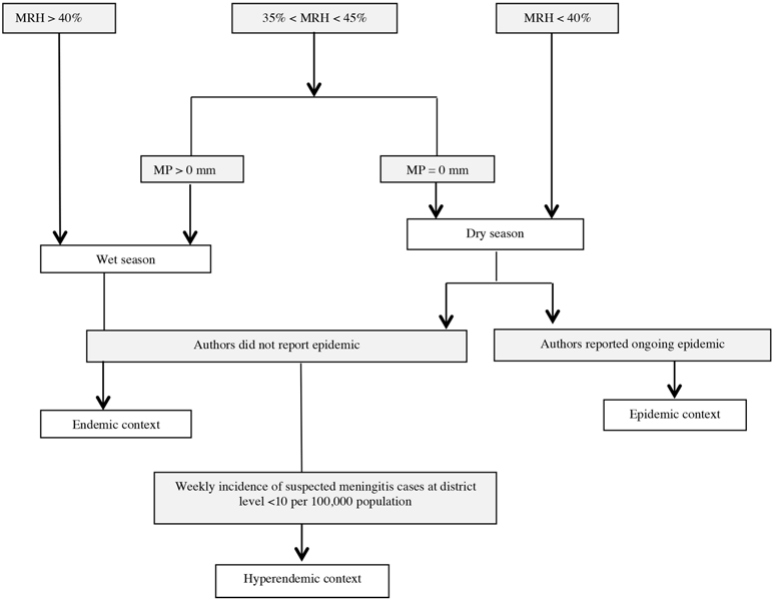
Algorithm for the definition of season and epidemiological context of case-carrier observation units reported by publications. MRH = Mean daily relative humidity in the two weeks preceding study onset or MRH of the study month (when only month of study was reported). MP: Mean daily precipitation amount (mm) during the two weeks preceding the study.

Eligible articles were scrutinized to identify additional information required, which then was sought from the articles’ authors, using data collection sheets. This concerned the number, over specific time periods, of confirmed Nm cases by serogroup, suspected case reporting and age-stratified data. A pair of incidence and carriage estimates during a given month in a given community was called “Case Carrier Observation Unit” (CCOU)” and was described by size of the surveyed population, carriage study sample size, serogroup-specific number of confirmed cases and carriers, and monthly incidence and carriage prevalence with measures of variance (standard errors or deviation). Each CCOU was categorised according to season (wet/dry) and epidemiological context (endemic, hyperendemic, or epidemic). The categorisation was conducted by two reviewers based on information provided by authors in the article, weekly incidence rates of suspected meningitis cases relating to the follow up period if available, and meteorological data as provided by authors or available on tutiempo.net following an algorithm (Fig. 1). Mean daily Relative Humidity (MRH) in the study area in the two weeks preceding study onset was the main criteria for season assignment. When only the month of study was reported, this was considered for MRH. Meteorological situations with MRH > 40% and MRH < 40% were defined wet and dry, respectively. If 35% < MRH < 45%, the mean precipitation (mm) during the two weeks preceding the study was taken into account. Within dry seasons with no reported epidemic, weekly incidence rates of suspected cases less than ten per 100,000 populations were classified as hyperendemic. [12, 13] Based on authors’ information, we assigned a causal serogroup to epidemics, and classified study populations as “vaccinated” if they have received a meningococcal mass vaccination against the relevant serogroup one week to three years prior to the study onset.

We evaluated the risk of bias in studies using the following criteria: (1) appropriateness of reported inclusion and exclusion criteria, (2) appropriateness of carriage study sampling design, (3) described bacterial identification protocol in accordance to World Health Organization (WHO) standards [14], (4) diagnostic criteria for meningitis diseased in accordance to WHO standards [15], (5) appropriateness of reported swabbing protocol, and (6) whether swabs were plated on site during population based carriage surveys.

Serogroup-specific case–carrier ratios (CCR) were computed for each CCOU as:

$$CCR=\frac{ncases/npopulation}{ncarriers/nsample}$$

Haldane’s continuity correction [16] was applied on CCOUs if cases, but no asymptomatic carriers have been identified. Using the Delta method [17], the variance of the natural logarithm of the CCR was calculated as:

$$Var=\frac{npopulation-ncases}{\left(npopulation)(ncases\right)}+\frac{nsample-ncarriers}{\left(npopulation)(ncases\right)}$$

Where *n* denotes numbers. For each epidemiological context, pooled serogroup-specific meningitis incidence, carriage prevalence, and CCR were estimated with 95% confidence intervals (95%-CI) using the inverse-variance random-effects model. This approach uses the inverse-variance weighting method to combine study-specific estimates into a weighted average estimate. Prior to combining study results, each study-specific estimate is weighed in inverse proportion to its variance. Inconsistency among CCRs of the same epidemiological context was quantified as the inconsistency index (I^2^): I^2^>50% was considered substantial heterogeneity and I^2^ <50% moderate inconsistency. The I^2^ statistics computed based on the Q statistics of the Cochran’s Q test has the advantage of not inherently depending on the number of studies included. Analyses were performed using STATA 11.2 (StataCorp LP) and The R foundation for statistical computation software v. 3.0.1.

## Results

**Figure 2:**
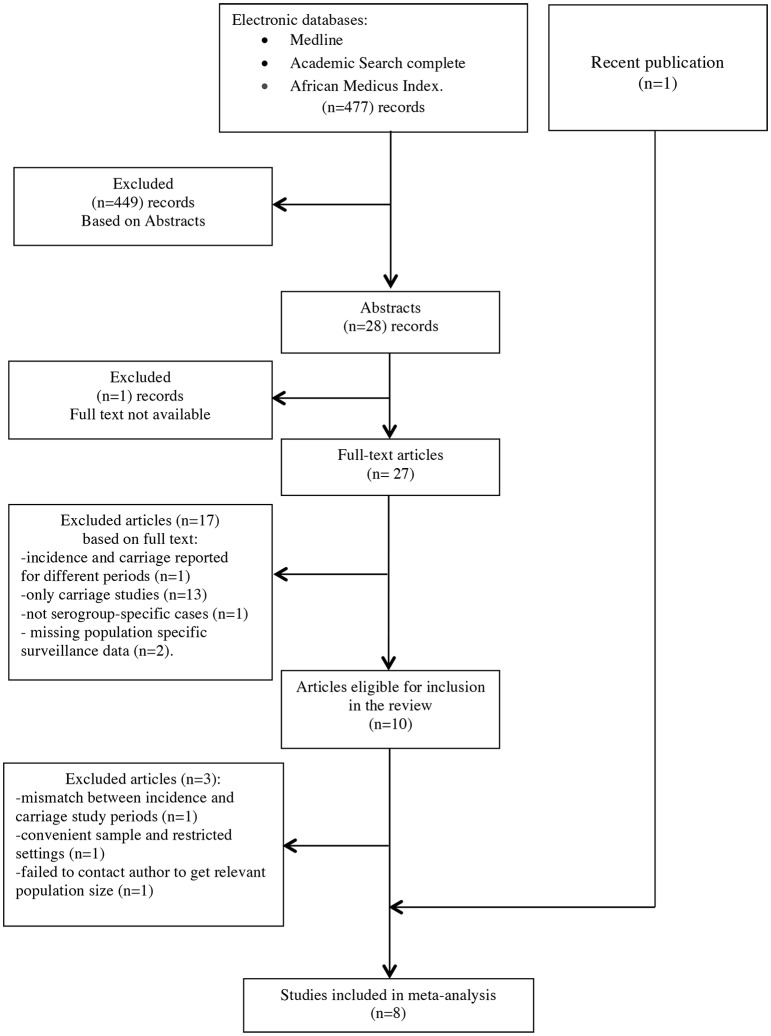
Flow diagram of study identification and inclusion in the systematic review on meningococcal case-carrier ratios in the African meningitis belt.

We retrieved 367 records from the initial search of which ten were eligible based on full text screening (Fig. 2). Three studies were excluded from the review because we failed to obtain information from authors on study population size [18], because the carriage study carried on a convenience sample [19], and because there was a mismatch between the time periods of meningitis surveillance and carriage survey, respectively. [20] The search update yielded 477 records with one recently published eligible study identified. [21] Overall, eight studies (Table 1) reporting 29 eligible CCOUs were available for meta-analysis on NmA, seven (27 CCOUs) on serogroup W and six (24 CCOUs) on serogroup X (S1 Table). Four studies were conducted in Burkina Faso [21–24] (eight CCOUs for NmA, eight for NmW), two in Niger [25, 26] (five for NmA, five for W, two for NmX) one in Ghana [10] (14 CCOUs for NmA, 14 for NmW, 14 for NmX) and one in the Gambia [11] (two CCOUs for NmA). One of the two NmA CCOUs in the Gambian study [11] was lately excluded from meta-analysis after contact with the main author, because neither requested information nor meteorological data was available to allow classification into the appropriate season and epidemiological context. For two studies [11, 24], confirmed cases in the hyperendemic context could only be obtained for 4- and 7-month periods, and we approximated monthly incidence as the average incidence. For NmA, four eligible CCOUs corresponded to the dry/epidemic context, 18 to the dry/hyperendemic context, and six to the wet/endemic context. For NmW, six CCOUs corresponded to wet/endemic context, and 21 to the dry/hyperendemic context. For NmX, six and 18 CCOUs corresponded to wet/endemic and dry/hyperendemic respectively.

Two studies [11, 25] had an unclear risk of bias with regards to their carriage study sampling design. One of these two studies was conducted in 1983 and was missing diagnostic criteria for meningitis cases. Another study [26] was subject to potential selection bias even though authors considered that the participants were representative of the target population (S1 Fig.). Age-specific estimates were accessible only for 7 CCOUs, all from studies conducted in Burkina Faso (three in epidemic context and four in hyperendemic context); in consequence, we did not conduct age-stratified analyses.

**Figure 3:**
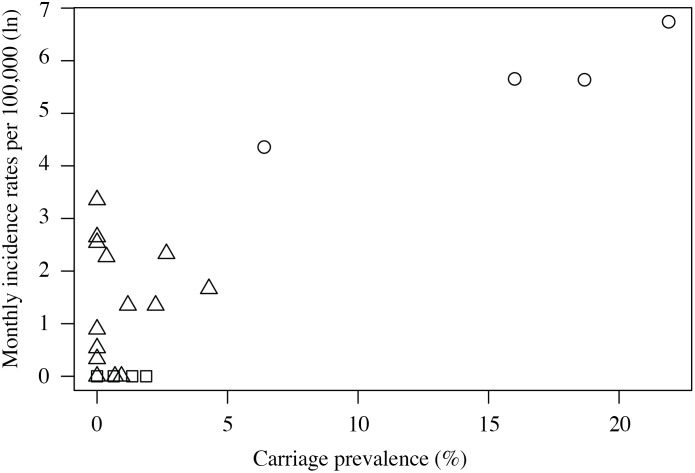
Scatterplot of meningococcal serogroup A monthly incidence rates and carriage prevalence across case carrier observation units. Squares show data points in endemic context; triangles show data points in hyperendemic context, and hallow circle show data points in epidemic context.

**Figure 4:**
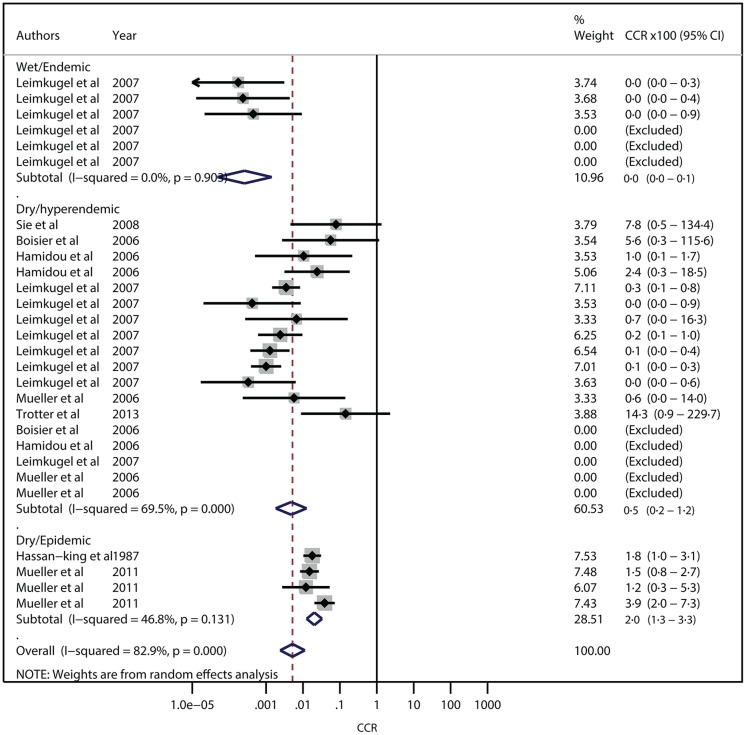
Forest plot for meta-analysis of serogroup A meningococcal meningitis case-carrier ratios according to epidemiological context in the African meningitis belt.

The pooled estimate of NmA carriage prevalence was similar in the endemic and hyperendemic context [0·53% (95%-CI, 0·09%–1·31%) and 0·50% (0·17%–0·98%), respectively], but 30-fold higher in the epidemic context [15·28% (8·58%–23·48%)]. Corresponding NmA meningitis monthly incidence rates per 100,000 were 0·17 (0·01–0·58), 2·64 (0·90–5·30) and 319 (150–549), respectively (Fig. 3). The resulting CCRs were 0·0×10^-2^ (0·0×10^-2^–0·1×10^-2^) for endemic, 0·5×10^-2^ (0·2×10^-2^–1·2×10^-2^) for hyperendemic, and 2·0×10^-2^ (1·3×10^-2^–3·3 ×10^-2^) for epidemic situations (Fig. 4). Heterogeneity between CCOUs was low for the endemic (I^2^ = 0·0%, *P* = 0·903), substantial for the hyperendemic (I^2^ = 69·5%, *P* = 0·000) and moderate for the epidemic context (I^2^ = 46·8%, *P* = 0·131).

The heterogeneity of the hyperendemic estimate was reduced by stratification by vaccination status (14 CCOUs were observed 1 week to 3 years after serogroup A meningococcal polysaccharide vaccine campaigns) (S2 Fig. and S3 Fig.). For the endemic situation, CCR was now 0·1×10^-2^ (95%-CI, 0·0×10^-2^–0·1×10^-2^; I^2^= 0·0%, *P*=0·903; N = 6) among vaccinated, while no data were available for unvaccinated populations. For the hyperendemic context, CCR was 0·2×10^-2^ (0·1×10^-2^–0·5×10^-2^; I^2^=37·9%, *P*=0·106; N = 14) among vaccinated and 8·8 ×10^-2^ (1·7×10^-2^–46·0×10^-2^; I^2^=0·0%, *P*=0.899; N = 4) for unvaccinated populations. For the epidemic context, CCR was 1·5×10^-2^ (0·8×10^-2^–2·7×10^-2^; N = 1) among vaccinated and 3·3 ×10^-2^ (1·2×10^-2^–4·4×10^-2^; I^2^=52·7%, *P*=0·120; N = 3) among unvaccinated populations. We could not identify any other factor of heterogeneity. For NmW, the pooled carriage prevalences in endemic and hyperendemic contexts were 0·15% (0·02–0·37%) and 1·08% (0·46–1·95%), respectively. Corresponding monthly incidence rates per 100,000 were 0·18 (0·01–0·58) and 0·73 (0·26–1·43), respectively. No carriage and incidence data was available for the epidemic context with serogroup W. The CCR was 0·0×10^-2^ (0·0×10^-2^–0·1×10^-2^ (only one CCOU provided information) and 0·1×10^-2^ (0·1×10^-2^–0·2×10^-2^; I^2^=37%, *P*=0·103) for endemic and hyperendemic contexts, respectively. No carriage and incidence data was available for the epidemic context.

Pooled carriage prevalence of NmX was 1·40% (0·07–4·34%) in the endemic and 0·78% (0·15–1·90%) in the hyperendemic context. Corresponding monthly incidence rates per 100,000 were 0·18 (0·01–0·58) and 0·19 (0·06–0·39), respectively. The resulting CCR was 0·0×10^-2^ (0·0×10^-2^–0·1×10^-2^; I^2^=7·4%, *P*=0·373) for the endemic context, and had an upper 95% confidence limit below 0·0005 for the hyperendemic context (the software did not specify the central estimate at the fourth decimal below 0·000). No carriage and incidence data was available for epidemic context with serogroup X.

## Discussion

This is the first study that systematically reviews and synthesizes available serogroup-specific incidence and carriage data of meningococcal meningitis in the meningitis belt. The substantially higher CCR during non-epidemic dry seasons, compared to wet season suggests that seasonal hyperendemicity of NmA meningitis appears related to an increased risk of meningitis given asymptomatic colonisation, but not related to an increase in transmission and colonisation. In contrast, the occurrence of NmA epidemics appears related to a substantial increase in meningococcal transmission and colonisation, and to a lesser extent with increased risk of meningitis given carriage. These results lend force to some hypotheses on the causation of seasonal hyperendemicity and epidemics and infirm others.

In pooled analyses, meningococcal carriage prevalence of NmA, NmW and NmX did not increase substantially from endemic (wet season) to hyperendemic context (dry season). NmW did show a significant difference, however, its magnitude (0.15% vs. 1.08%) probably is not important from a biological standpoint: using a recently published model for meningococcal meningitis epidemics [27], for a fixed rate of progression from carriage to disease, seasonal oscillations of disease incidence with magnitudes as observed (10–100-fold) could be produced by seasonal variations of carriage prevalence between <1% and 40%. A review of carriage studies in the meningitis belt concluded that changes in the prevalence of carriage are not linked to season in any consistent way. [28] Minor variations have been described in series of cross-sectional studies [29], but should not be interpreted as systematic seasonal variation. They likely correspond to long-term strain variations rather than a seasonal phenomenon. In consequence, seasonal differences in bacterial transmission e.g. mediated by improved pathogen survival [30] or different social mixing patterns, should be dismissed as explanation for seasonality of meningococcal meningitis. [31]

Statistical analyses only allowed an approximation of fold-increase in CCR from wet to dry season between >5 to infinite. This was due to endemic incidences being close to zero, with an endemic CCR of 0.00. Given that carriage prevalence was the same for endemicity and hyperendemicity, but incidence differed 15-fold, we can assume the increase in CCR being around 15-fold. Meteorological modelling studies suggest that relative humidity below 40% in combination with high aerosol load strongly correlates with hyperendemicity of meningococcal meningitis in the meningitis belt. [32] No demonstrated pathophysiological explanation exists on how dry and dusty air can facilitate meningitis, but it could be intuitive that such exposure can weaken the nasopharyngeal mucosa and therefore facilitate meningococcal invasion into tissues and bloodstream. Meningococcal septicaemia is rarely observed in the meningitis belt, suggesting that facilitated meningococcal invasion may not typically involve invasion into the blood stream. In addition, meningococcal invasion of olfactory nerve structures mounting towards the meninges has been found in mice. [33] In this scenario, environmental damage of the mucosa would lead to facilitated direct meningeal invasion by meningococci. In theory, increased meningitis incidence also could be attributed to reduced immune function during the dry season, but no data are available to inform this hypothesis. In any case, this around 15-fold seasonal increase in invasion is one of the strongest impacts that usual meteorological variations have on health. Upcoming climate changes may increase the proportion of the world’s population exposed to such prolonged dry seasons and high aerosol load, and may increase the resulting global burden of disease. Pneumococcal meningitis, a major cause of morbidity and mortality in the African meningitis belt, also shows a 10-fold increase in incidence during dry seasons, [34] and similar mechanisms may be involved. Measures to prevent this seasonally increased risk of invasive disease given asymptomatic bacterial infection could be developed, in addition to pathogen-specific vaccines.

As opposed to constant NmA carriage between endemicity and hyperendemicity, we found 30-fold increased NmA carriage prevalence during epidemics, which may be causal for, or a consequence of epidemics. Meningitis patients do not transmit meningococci substantially more frequently than healthy persons, as disease-specific spreading behaviour such as vomiting occurs after disease onset, when patients are already bound to bed. It is therefore more likely that increased acquisition and transmission contribute to the occurrence of epidemics. If the dry season environment greatly facilitated invasion of colonising meningococci, an increase in colonisation would simply lead to proportionally increased meningitis incidence. However, the estimated 30-fold increase in NmA carriage prevalence suggests that the carriage increase is not sufficient to explain on its own the 130-fold increase in incidence, as postulated in the hypothetical model by Mueller & Gessner. According to our results, a further slight increased risk of invasion given colonisation occurs during epidemics (4-fold increase in CCR). Respiratory virus infections could play such a double role, as they probably facilitate meningococcal adhesion to the mucosa or increase transmission via coughing and sneezing, and also temporarily reduce immune defence against bacterial disease by disrupting the immune response against encapsulated bacteria. [35] This is supported by observations during NmA meningococcal epidemics, where carriage was associated with respiratory infection symptoms [36, 37] and participants reporting recent flu-like symptoms were at increased risk of subsequently presenting with confirmed or purulent meningitis. [23]

Although the hypothetical model by Mueller&Gessner concentrated on climatic factors to explain the variation between endemic and hyperendemic situation, in principal, seasonal variations of viral co-infections, or other intermediary factors, could contribute to increase risk of meningococcal invasion (but not transmission, given our results)

Our analyses stratifying by vaccination status suggest that polysaccharide vaccination against serogroup A related to a reduced risk of meningitis given colonisation, possibly more in hyperendemic (where there was a significant different in CCR between vaccinated and unvaccinated populations) than epidemic situations. However, interpretation by epidemiological situations may be inappropriate due to the small number of relevant observations for unvaccinated populations and potential heterogeneity between studies.

We cannot provide clear evidence on the question whether NmW behaves similar to NmA, as no data for the epidemic context were available. Both incidence and CCR increased from endemic to hyperendemic context, although to lesser extent than NmA. We did not observe a clear seasonality for NmX meningitis. Leimkugel et al. observed periods of substantially increased NmX carriage during hyperendemicity (prevalence 17%), but outside epidemics, NmX meningitis incidence usually remained low at levels comparable to endemic periods of NmA and NmW. The risk of meningitis given colonisation appears to be substantially lower compared to NmA. [10] It is unclear whether this is due to better natural immunity or a lesser capacity for invasion. Combined carriage and surveillance studies during periods with strong serogroup X or W incidence and epidemics are needed to better understand the epidemic behaviour of these serogroups.

There are some limitations to our analysis. The estimated CCRs are imprecise, as surveillance systems unlikely achieve complete case identification and carriage studies probably underestimate colonisation prevalence. [38] Furthermore, except for one study performing repeated assessments, [10] we cannot follow the CCR variation of incidence-carriage pairs across epidemiological contexts, but are limited to group comparison. Methodological differences between studies may have led to over- or underestimating CCR changes between epidemiological contexts; e.g. the series of CCOUs reported by Leimkugel et al. [10] generally showed lower CCR. Finally, we did not analyse age-specific CCRs, due to difficulties in re-analysing original data collected up to 20 years ago. Such age stratification would provide insight into the high incidence among teenagers, but its omission unlikely biases our results. In this study, we cannot evaluate the association between specific meteorological features, such as humidity or aerosol load, and changes in meningococcal meningitis epidemiology. To identify the mechanism through which dry season is associated with higher meningitis incidence, correlation studies between meteorological and incidence data are more appropriate.

The most important limitation is that we transfer results from an ecological analysis to the individual level of susceptibility for disease, which will only be a further step in evaluating a hypothesis and does not have the validity of clinical evidence.

In conclusion, this study provides orientation on how risk of bacterial invasion and transmission or colonisation may interact to produce the particular epidemiology of the African meningitis belt. The findings will be useful for developing models to evaluate vaccination strategies, to develop further relevant research. They leave room to hypothesis that other diseases, such as pneumococcal meningitis and pneumonia, may be concerned by a complex interaction between climatic environment, bacteria, potentially co-infections, and human mucosal and immune defence.

## Supporting Information

S1 PRISMA Checklist PRISMA 2009 checklist. (DOC)

S1 Fig Risk of bias summary for included studies. (TIF)

S2 Fig Forest plot for meta-analysis of serogroup A meningococcal meningitis case-carrier ratios in vaccinated populations according to epidemiological context in the African meningitis belt. (TIF)

S3 Fig Forest plot for meta-analysis of serogroup A meningococcal meningitis case-carrier ratios in unvaccinated populations according to epidemiological context in the African meningitis belt. (TIF)

S1 Table Summary of serogroup-specific Case Carrier Observation Unit by epidemiologic context. (DOCX)

S1 Text Protocol for Systematic Review and Meta-analysis. (PDF)

S2 Text Search Strategy. (DOCX)
