# Supplementary material for: Incidence, Carriage and Case-Carrier Ratios for Meningococcal Meningitis in the African Meningitis Belt: A Systematic Review and Meta-Analysis
Source: PLoS One. 2015 Feb 6;10(2):e0116725. doi: 10.1371/journal.pone.0116725 (PMC4319942; doi:10.1371/journal.pone.0116725)
Supplement: S1 PRISMA Checklist — (DOC) [file pone.0116725.s001.doc]

| **Section/topic** | **#** | **Checklist item** | **Reported on page #** |
| --- | --- | --- | --- |
| **TITLE** | | |  |
| Title | 1 | Incidence, carriage and case-carrier ratios for meningococcal meningitis in the African meningitis belt: a systematic review and meta-analysis. | 1 |
| **ABSTRACT** | | |  |
| Structured summary | 2 | Background: To facilitate the interpretation of meningococcal meningitis epidemiology in the "African meningitis belt", we aimed at obtaining serogroup-specific pooled estimates of incidence, carriage and case-carrier ratios for meningococcal meningitis in the African meningitis belt and describe their variations across the endemic, hyperendemic and epidemic context.  Methods: We conducted a systematic review and meta-analysis of studies reporting serogroup-specific meningococcal meningitis monthly incidence and carriage in the same population and time period. Epidemiological contexts were defined as endemic (wet season, no epidemic), hyperendemic (dry season, no epidemic), and epidemic (dry season, epidemic).  Findings: Eight studies reporting a total of eighty pairs of serogroup-specific meningococcal meningitis incidence and carriage estimates were included in this review. For serogroup A, changes associated with the transition from endemic to hyperendemic incidence and from hyperendemic to epidemic incidence were 15-fold and 120-fold respectively. Changes in carriage prevalence associated with both transitions were 1-fold and 30-fold respectively.  For serogroup W and X, the transition from endemic to hyperendemic incidence involved a 4-fold and 1·1-fold increase respectively. Increases in carriage prevalence for the later transition were 7-fold and 1·7-fold respectively. No data were available for the hyperendemic-epidemic transition for these serogroups. Our findings suggested that the regular seasonal variation in serogroup A meningococcal meningitis incidence between the rainy and the dry season could be mainly driven by seasonal change in the ratio of clinical cases to subclinical infections. In contrast appearance of epidemic incidences is related to a substantial increase in transmission and colonisation and to lesser extent with changes in the case-carrier ratio.  Conclusion: Seasonal change in the rate of progression to disease given carriage together with variations in frequency of carriage transmission should be considered in models attempting to capture the epidemiology of meningococcal meningitis and mainly to predict meningitis epidemics in the African meningitis belt. | 2 |
| **INTRODUCTION** | | |  |
| Rationale | 3 | Factors leading to the occurrence of meningococcal meningitis epidemics in the African meningitis belt remain hypothetic. There is need for better understanding the meningococcal epidemics process as it occurs in the African meningitis belt in order to better design control strategies including vaccination. | 4 |
| Objectives | 4 | To provide best evidence on the magnitude of variation of meningococcal serogroup specific disease incidence, carriage prevalence and case-carrier ratio according to the endemic (wet season, no epidemic), hyperendemic (dry season, no epidemic) and epidemic (dry season, epidemic) epidemiological context and how it relates to the epidemiology of meningococcal meningitis in the African meningitis belt. | 5 |
| **METHODS** | | |  |
| Protocol and registration | 5 | This review was based on a writing protocol, which can be made available by the corresponding author upon request. | 5 |
| Eligibility criteria | 6 | Population based studies reporting meningococcal serogroup-specific cases and carriers observations in the same population and time period were considered in this review. We included studies conducted after 1969 when the distinction between *N. meningitidis* and *N. lactamica* was possible. Studies published in language English, French, or German were eligible. | 5, 6,7 |
| Information sources | 7 | Describe all information sources (e.g., databases with dates of coverage, contact with study authors to identify additional studies) in the search and date last searched. | 5 |
| Search | 8 | Search strategy : Medline via EBSCOhost research platform  **#1)** SH Meningitis, Meningococcal  **#2)** TI (Meningitis, Meningococcal, Serogroup Y) or TI (Serogroup Y, Meningococcal Meningitis) or TI (Meningococcal Meningitis, Serogroup Y) or TI (Meningitis, Meningococcal, Serogroup C) or TI (Serogroup C Meningococcal Meningitis) or TI (Meningococcal Meningitis, Serogroup C)  **#3)** TI (Meningitis, Meningococcal, Serogroup B) or TI (Serogroup B Meningococcal Meningitis) or TI (Meningococcal Meningitis, Serogroup B)  **#4)** TI (Meningitis, Meningococcal, Serogroup A) or TI (Serogroup A Meningococcal Meningitis) or TI (Meningococcal Meningitis, Serogroup A)  **#5)** TI (Meningococcal Meningitis, Serogroup W 135) or TI (Serogroup W-135, Meningococcal Meningitis) or TI (Serogroup W 135)  **#6)** TI (Meningitis, Meningococcal, Serogroup X) or TI (Serogroup X Meningococcal Meningitis) or TI (Meningococcal Meningitis, Serogroup X)  **#7)** (#2 or #3 or #4 or #5 or #6)  **#8)** AB (Meningitis, Meningococcal, Serogroup X) or AB (Serogroup X Meningococcal Meningitis) or AB (Meningococcal Meningitis, Serogroup X)  **#9)** AB (Meningitis, Meningococcal) or AB (Meningococcal Meningitis) or AB (Neisseria meningitis) or AB (Meningitis, Cerebrospinal) or AB (Acute meningitis) or AB (Epidemic meningitis) or AB (Meningitis, Meningococcic)  **#10)** TI (Meningitis, Meningococcal) or TI (Meningococcal Meningitis) or TI (Neisseria meningitis) or TI (Meningitis, Cerebrospinal) or TI (Acute meningitis) or TI (Epidemic meningitis) or TI (Meningitis, Meningococcic)  **#11)** AB (Meningitis, Meningococcal, Serogroup Y) or AB (Serogroup Y, Meningococcal Meningitis) or AB (Meningococcal Meningitis, Serogroup Y) or AB (Meningitis, Meningococcal, Serogroup C) or AB (Serogroup C Meningococcal Meningitis) or AB (Meningococcal Meningitis, Serogroup C)  **#12)** AB (Meningococcal Meningitis, Serogroup W 135) or AB (Serogroup W-135, Meningococcal Meningitis) or AB (Serogroup W 135) or  **#13)** AB (Meningitis, Meningococcal, Serogroup A) or AB (Serogroup A Meningococcal Meningitis) or AB (Meningococcal Meningitis, Serogroup A)  **#14)** AB (Meningitis, Meningococcal, Serogroup B) or AB (Serogroup B Meningococcal Meningitis) or AB (Meningococcal Meningitis, Serogroup B)  **#15)** (#8 or #9 or #10 or #11 or #12 or #13 or #14)  **#16)** #7 or #15  **#17)** #1 and #16  **#18)** MH Africa/ or MH African meningitis belt/ or MH meningitis belt/ or MH Africa south of the Sahara/ or MH sub-Saharan Africa / or MH Burkina Faso/ or MH Niger/ or Niamey/ or MH Mali/ or MH Togo/ or MH Ghana/ or MH Côte d’Ivoire/ or MH Ivory Coast/ or MH Senegal/ or MH Chad/ or MH Ethiopia/ or MH Sudan/ or MH Benin/ or MH Nigeria/ or MH Cameroun/ or MH The Gambia/ or MH Gambia/ 26  **#19)** #17 and #18 | 5,6 |
| Study selection | 9 | Studies were first screened based on title and abstract by one reviewer. Studies, which successfully passed the initial screening, underwent a full text screening using inclusion criteria defined in the review protocol. Two reviewers conducted selection of studies based on full text screening. | 5, 6 |
| Data collection process | 10 | Data collection was conducted based on a piloted form by one reviewer and crosschecked by another reviewer. We contacted authors of two studies to confirm data or to get additional information. We used Graph Extract v2.5 to data extraction from two studies. | 6 |
| Data items | 11 | A pair of incidence and carriage estimates during a given month in a given community was called “Case and Carrier Observation Unit” (CCOU)” and we collect data on size of the surveyed population, carriage study sample size, serogroup-specific number of confirmed cases and carriers, and monthly incidence and carriage prevalence with measures of variance (standard errors or deviation) when reported. Based on an algorithm, each CCOU was categorised into one of the following epidemiological context: endemic (wet season, no epidemic), hyperendemic (dry season, no epidemic) and epidemic (dry season, epidemic). | 6 |
| Risk of bias in individual studies | 12 | We evaluated the risk of bias in studies using the following criteria: (1) appropriateness of reported inclusion and exclusion criteria, (2) appropriateness of carriage study sampling design, (3) described bacterial identification protocol in accordance to World Health Organization (WHO) standards15, (4) diagnostic criteria for meningitis diseased in accordance to WHO standards16, (5) appropriateness of reported swabbing protocol, and (6) whether swabs were plated on site during population based carriage surveys.  For each criterion, study was classified as low risk of bias or high risk of bias based on information reported in the paper. When judgement cannot be directly made based on information provided in the paper or information was missing and study was too old to ask the author for details we considered those studies at unknown risk of bias with regard to the criteria being evaluated. | 7 |
| Summary measures | 13 | Summary measures were, serogroup-specific meningococcal meningitis monthly incidence and carriage prevalence, and the case- carrier ratio. | 7 |
| Synthesis of results | 14 | Meta-analysis was conducted according to defined epidemiological context of the studies. Pooled serogroup-specific meningitis incidence, carriage prevalence, and case-carrier ratio were estimated with 95% confidence intervals (95%-CI) using the inverse-variance random-effects model. Inconsistency across individual study estimates was measured using the inconsistency index (I^2^): I^2^>50% was considered substantial heterogeneity and I^2^ <50% moderate inconsistency. | 7 |

Page 1 of 2

| **Section/topic** | **#** | **Checklist item** | **Reported on page #** |
| --- | --- | --- | --- |
| Risk of bias across studies | 15 | We did not evaluate the risk of bias across studies. |  |
| Additional analyses | 16 | We conducted subgroup analysis by comparing groups of studies in which the study target population had received meningococcal polysaccharide vaccine in the two weeks to three years preceding the study onset. | 9 |
| **RESULTS** | | |  |
| Study selection | 17 | 477 studies were retrieved from the search out of which eight met the inclusion criteria. The eight studies reported a total of eighty cases and carriers’ observations estimates. Studies were mainly excluded because they did not report both carriage prevalence and incidence data on the same target population over the same time period or they were primarily conducted on a convenient sample . | 8 |
| Study characteristics | 18 | For each study, we collected the following information. Study first author and publication year, location, design, sample size of participants, Study duration/ follow up period, season and epidemiological context of the study. | 8 |
| Risk of bias within studies | 19 | 5 out of the 8 studies included in this review were considered at low risk of bias. Two studies were considered at unknown risk of bias, because information for judgement was incomplete or missing from the publication. The carriage survey of one study was considered at high risk of bias because sampling was likely subject to coverage bias, even though the authors stated that the study sample is fairly representative of the target population. | 9 |
| Results of individual studies | 20 | Results for individual studies including meta-analysis results for serogroup A are provided in figure4 (Forest plot). For serogroups W and X, incidence and carriage estimates are reported for individual studies in table S1. | 9 |
| Synthesis of results | 21 | Meta-analysis of serogroup A carriage prevalence was similar in the endemic and hyperendemic context [0·53% (95%-CI, 0·09%–1·31%) and 0·50% (0·17%–0·98%), respectively], but 30-fold higher in the epidemic context [15·28% (8·58%–23·48%)]. Corresponding serogroup A meningitis monthly incidence rates per 100,000 were 0·17 (0·01–0·58), 2·64 (0·90–5·30) and 319 (150–549), respectively (Figure 3). The resulting CCRs were 0·0x10-2 (0·0x10-2–0·1x10^-2^), 0·5x10^-2^ (0·2x10^-2^–1·2x10- 2) and 2·0x10-2 (1·3x10^-2^–3·3 x10^-2^), respectively (Figure 4). Heterogeneity between CCOUs was low for the endemic (I2= 0·0% *P*=0·903), substantial for the hyperendemic (I2= 69·5% *P*=0·000) and moderate for the epidemic context (I2 = 46·8%, *P*=0·131).  For serogroup W, the pooled carriage prevalences in endemic and hyperendemic contexts were 0·15% (0·02–0·37%) and 1·08% (0·46–1·95%), respectively. Corresponding monthly incidence rates per 100,000 were 0·18 (0·01–0·58) and 0·73 (0·26–1·43), respectively. No carriage and incidence data was available for the epidemic context with serogroup W.  The CCR was 0·0x10-2 (0·0x10-2–0·1x10^-2^ (only one CCOU provided information) and 0·1x10^-2^ (0·1x10^-2^–0·2x10^-2^; I2=37%, *P*=0·103) for endemic and hyperendemic contexts, respectively. No carriage and incidence data was available for the epidemic context.  For NmX, pooled carriage prevalence was 1·40% (0·07–4·34%) in the endemic and 0·78% (0·15–1·90%) in the hyperendemic context. Corresponding monthly incidence rates per 100,000 were 0·18 (0·01–0·58) and 0·19 (0·06–0·39), respectively. The resulting CCR was 0·0x10-2 (0·0x10-2–0·1x10^-2^; I2=7·4%, *P*=0·373) for the endemic context, and had an upper 95% confidence limit below 0·0005 for the hyperendemic context (the software did not specify the central estimate at the fourth decimal below 0·000). No carriage and incidence data was available for epidemic context with serogroup X. | 9, 10 |
| Risk of bias across studies | 22 | We didn’t access risk of bias across studies. |  |
| Additional analysis | 23 | For meta-analysis of serogroup A CCR, the heterogeneity of the hyperendemic estimate was reduced to I2=37·9% (*P*=0·106) if only studies performed in vaccinated populations were included (14 CCOUs) (Appendix 2, Figure 5). The corresponding CCR was 0·1x10^-2^ (95%-CI, 0·0x10-2–0·1x10^-2^; I2= 0·0% *P*=0·903) for the endemic, and 0·2x10^-2^ (0·1x10^-2^–0·5x10^-2^) for the hyperendemic context. There was only one CCOU among a vaccinated population for the epidemic context, with a CCR of 1·5x10^-2^ (0·8x10^-2^–2·7x10^-2^). Restricting CCR meta-analysis to non-vaccinated populations yielded CCR of 8·8x10^-2^ (1·7 x10^-2^ –46 x10-2; I2 =0·0% *P*=0.899) and 3·3 x10^-2^ (1·2 x10^-2^ –4·4 x10^-2^; I2 =52·7% *P*=0·120) for hyperendemic and epidemic contexts, respectively, and no data for the endemic context. We could not identify any other factor of heterogeneity. | 10 |
| **DISCUSSION** | | |  |
| Summary of evidence | 24 | The ubiquous seasonal increase of serogroup A or W meningitis cases in the African meningitis belt appears related to an increased risk of invasive disease given carriage, and not to an increase in transmission or colonisation. By contrast, the occurrence of localised serogroup A epidemics appears related to a substantial increase in transmission and colonisation, and to a lesser extent with increased risk of meningitis given carriage. Measures to prevent this seasonally increased risk of invasive disease given asymptomatic infection should be developed, in addition to pathogen-specific vaccines. Models attempting to predict meningitis epidemics and vaccination impact in the meningitis belt should allow for seasonal change in the rate of progression from carrier state to disease as well as change in carriage transmission. | 10,11,12,13 |
| Limitations | 25 | This review used an ecological proxy: the case-carrier ratio (CCR) to understand how susceptibility to meningococcal meningitis given carriage varies according to season and epidemiological contexts in the meningitis belt. Any conclusion on individual susceptibility for invasive disease given colonisation is limited. Also, the estimated CCRs may be imprecise, as surveillance systems may not achieve complete case identification and carriage studies probably underestimate colonisation prevalence. Furthermore, except for one study performing repeated assessments [11], we cannot follow the CCR variation of incidence-carriage pairs across epidemiological contexts, but are limited to group comparison. Methodological differences between studies may have led to over- or underestimating CCR changes between epidemiological contexts; e.g. the series of CCOUs reported by Leimkugel11 usually showed lower CCR. Finally, we did not analyse age-specific CCRs, due to difficulties in re-analysing original data collected up to 20 years ago. Such age stratification would provide insight into the high incidence among teenagers, but its omission unlikely biases our results. | 14 |
| Conclusions | 26 | Seasonal increase of serogroup A or W meningitis cases as it occurs in the meningitis belt appears related to an increased risk of invasive disease given carriage, and not to an increase in transmission or colonisation. By contrast, the occurrence of localised serogroup A epidemics appears related to a substantial increase in transmission and colonisation, and to a lesser extent with increased risk of meningitis given carriage. Measures to prevent this seasonally increased risk of invasive disease given asymptomatic infection should be developed, in addition to pathogen-specific vaccines.  While this review provides essential input for the understanding of the meningitis belt phenomenon, future carriage studies should be systematically combined with meningitis surveillance, be repeated in endemic, hyperendemic and epidemic contexts, and regularly pooled with comparable studies. A particular need appears for such evaluation during epidemics due to serogroups W or X. | 15 |
| **FUNDING** | | |  |
| Funding | 27 | The study did not receive any specific funding. TK was financially supported by EHESP School of Public Health during a Master internship and by Université Pierre et Marie Curie (Paris 6) through a doctoral contract. |  |

*From:*  Moher D, Liberati A, Tetzlaff J, Altman DG, The PRISMA Group (2009). Preferred Reporting Items for Systematic Reviews and Meta-Analyses: The PRISMA Statement. PLoS Med 6(6): e1000097. doi:10.1371/journal.pmed1000097

For more information, visit: **www.prisma-statement.org**.

Page 2 of 2
