# Supplementary figures and images for: Incidence, Carriage and Case-Carrier Ratios for Meningococcal Meningitis in the African Meningitis Belt: A Systematic Review and Meta-Analysis
Source: PLoS One. 2015 Feb 6;10(2):e0116725. doi: 10.1371/journal.pone.0116725 (PMC4319942; doi:10.1371/journal.pone.0116725)

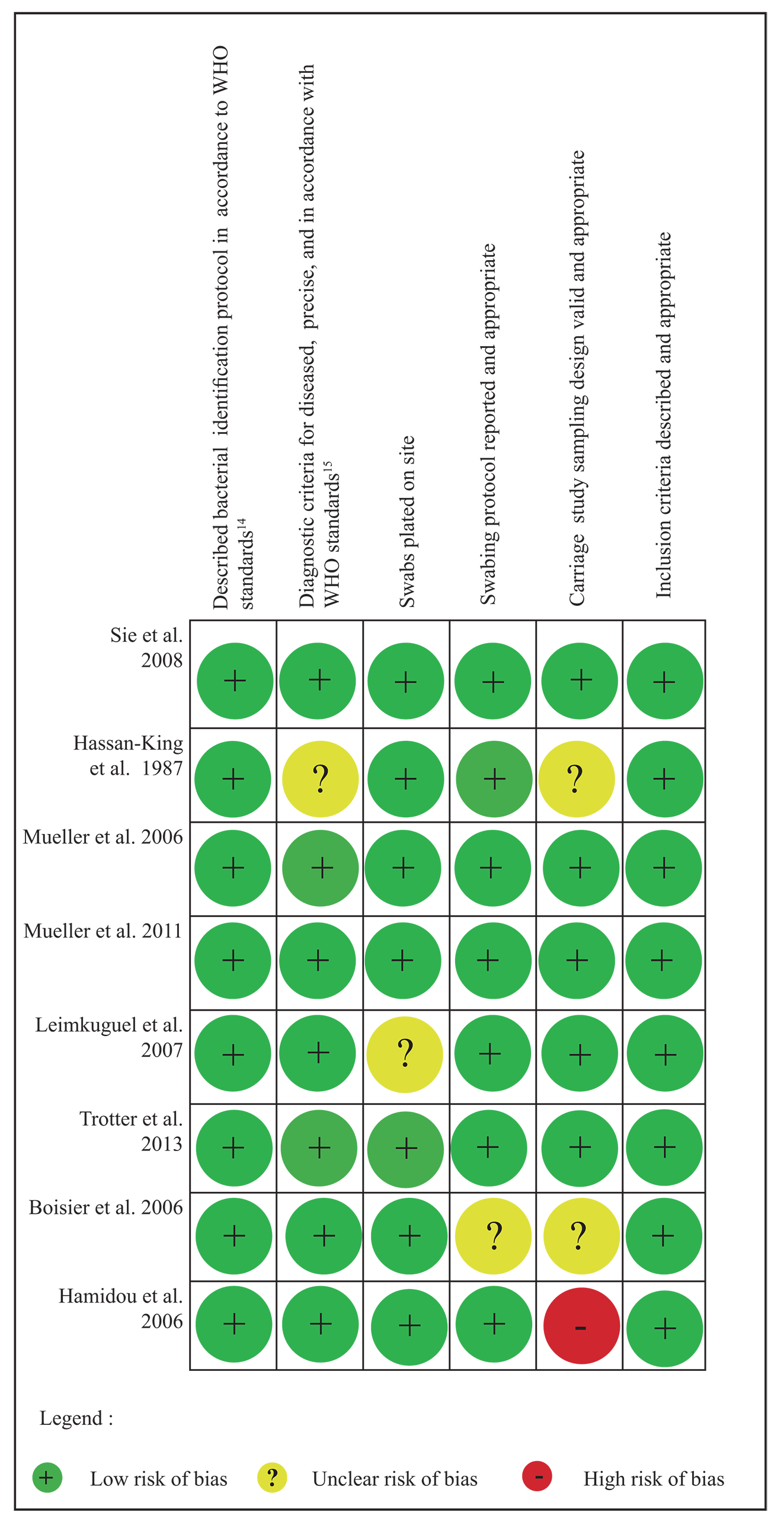

Supplement: S1 Fig — (TIF) [file pone.0116725.s002.tif]

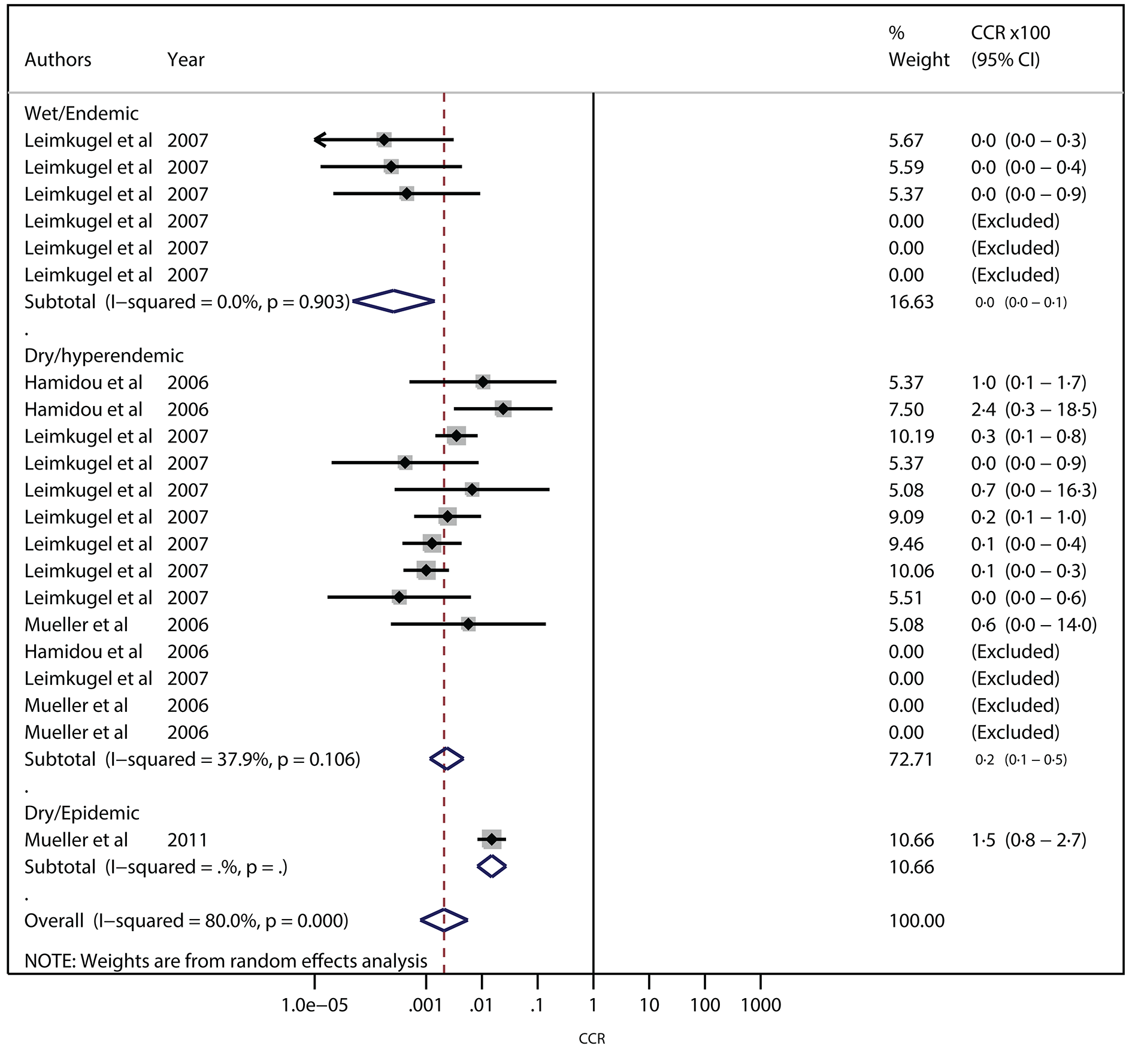

Supplement: S2 Fig — (TIF) [file pone.0116725.s003.tif]

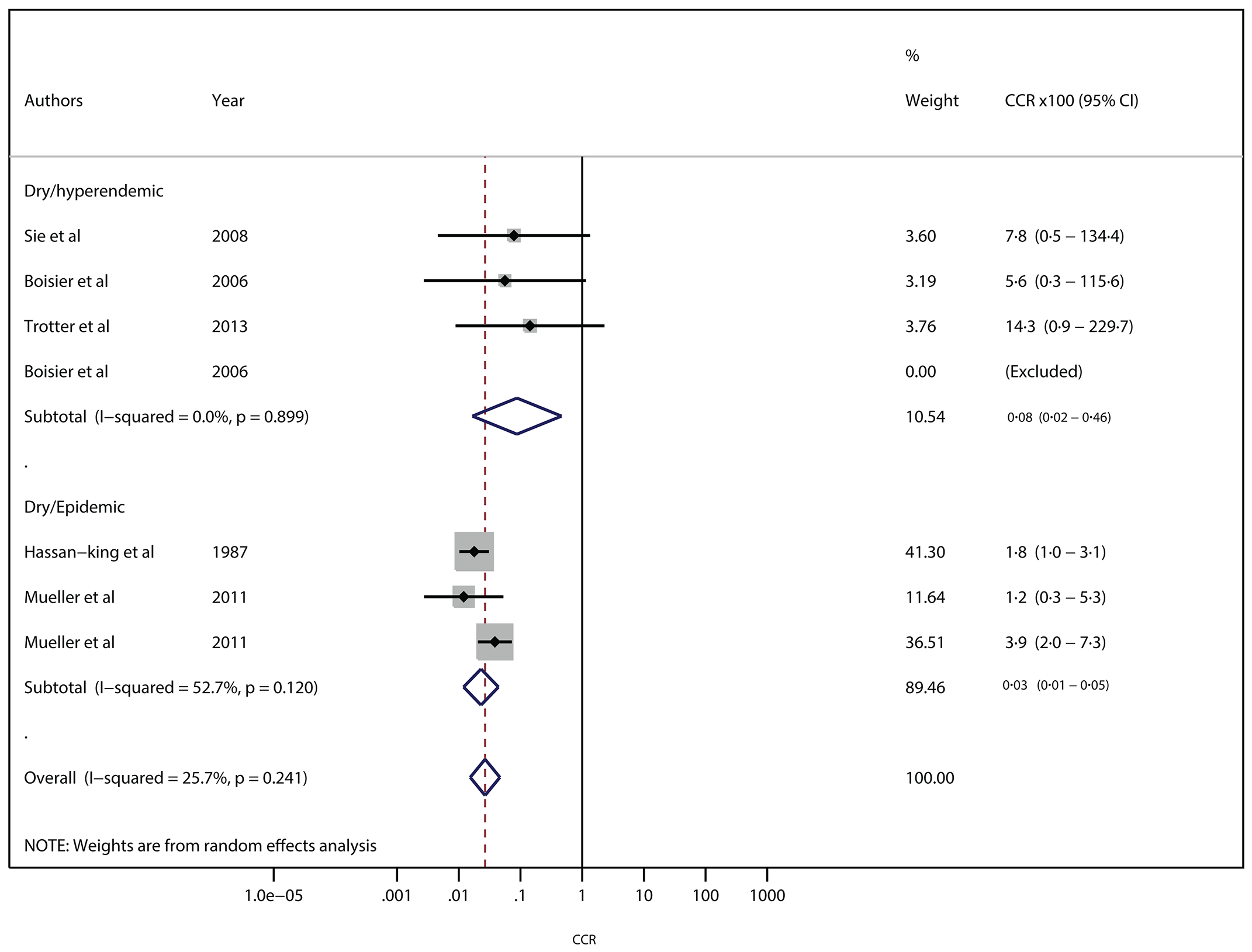

Supplement: S3 Fig — (TIF) [file pone.0116725.s004.tif]
