# Supplementary material for: Incidence, Carriage and Case-Carrier Ratios for Meningococcal Meningitis in the African Meningitis Belt: A Systematic Review and Meta-Analysis
Source: PLoS One. 2015 Feb 6;10(2):e0116725. doi: 10.1371/journal.pone.0116725 (PMC4319942; doi:10.1371/journal.pone.0116725)
Supplement: S1 Table — (DOCX) [file pone.0116725.s005.docx]

**Table S1. Summary of serogroup-specific Case Carrier Observation Unit by epidemiologic context.**

| **Authors. Publication Year [Reference]** | **Study**  **Month-Year** | **Monthly incidence** | | **Carriage prevalence** | |
| --- | --- | --- | --- | --- | --- |
|  |  | **cases/N** | **incid/100,000 pop** | **carriers/n** | **(%)** |
| **Endemic/Wet, Serogroup A** |  |  |  |  |  |
| Leimkugel et al. 2007 [10] | Nov-2004 | 0/140000 | 0·0 | 2/313 | 0·64 |
| Leimkugel et al. 2007 | Nov-2002 | 0/140000 | 0·0 | 6/319 | 1·88 |
| Leimkugel et al. 2007 | Nov-2005 | 0/140000 | 0·0 | 0/334 | 0·00 |
| Leimkugel et al. 2007 | Nov-2003 | 0/140000 | 0·0 | 4/297 | 1·35 |
| Leimkugel et al. 2007 | Nov-2000 | 0/140000 | 0·0 | 0/301 | 0·00 |
| Leimkugel et al. 2007 | Nov-2001 | 0/140000 | 0·0 | 0/306 | 0·00 |
|  |  |  |  |  |  |
| **Hyperendemic/Dry, Serogroup A** |  |  |  |  |  |
| Boisier et al. 2006 [25] | May-2003 | 2/7237 | 27·6 | 0/80 | 0·00 |
| Boisier et al. 2006 | Feb-2004 | 0/7469 | 0·0 | 0/70 | 0·00 |
| Hamidou et al. 2006 [26] | Feb-2003 | 2/138057 | 1·4 | 0/287 | 0·00 |
| Hamidou et al. 2006 | Mar-2003 | 12/138057 | 8·7 | 1/277 | 0·36 |
| Hamidou et al. 2006 | May-2003 | 0/138057 | 0·0 | 0/272 | 0·00 |
| Leimkugel et al. 2007 | Apr-2002 | 4/140000 | 2·9 | 4/339 | 1.18 |
| Leimkugel et al. 2007 | Apr-2001 | 1/140000 | 0·7 | 0/310 | 0·00 |
| Leimkugel et al. 2007 | Apr-2004 | 6/140000 | 4·3 | 15/350 | 4·28 |
| Leimkugel et al. 2007 | Apr-1998 | 13/140000 | 9·3 | 8/301 | 2·65 |
| Leimkugel et al. 2007 | Apr-2000 | 0/140000 | 0·0 | 0/298 | 0·00 |
| Leimkugel et al. 2007 | Apr-2005 | 0/140000 | 0·0 | 3/321 | 0·93 |
| Leimkugel et al. 2007 | Apr-2003 | 4/140000 | 2·9 | 7/312 | 2·24 |
| Leimkugel et al. 2007 | Apr-1999 | 0/140000 | 0·0 | 2/292 | 0·68 |
| Mueller et al. 2006 [22] | Apr-2003 | 0/253605 | 0·0 | 0/469 | 0·00 |
| Mueller et al. 2006 | Mar-2003 | 1/253605 | 0·4 | 0/482 | 0·00 |
| Mueller et al. 2006 | Feb-2003 | 0/253605 | 0·0 | 0/448 | 0·00 |
| Sié et al. 2008 [24] | Apr-2006 | 9/76847 | 11·7 | 0/316 | 0·00 |
| Trotter et al. 2013 [21] | 28th Feb –7th Mar -2008 | 82/623303 | 13·1 | 0/538 | 0·00 |
|  |  |  |  |  |  |
| **Epidemic/Dry, Serogroup A** |  |  |  |  |  |
| Hassan-King et al. 1987 [11] | Jan–Apr-1983 | 37/13000 | 284·6 | 16/100 | 16·00 |
| Mueller et al. 2011 [23] | Mar-2006 | 13/4640 | 280·2 | 59/316 | 18·67 |
| Mueller et al. 2011 | Mar- 2006 | 2/2600 | 76·9 | 13/203 | 6·40 |
| Mueller et al. 2011 | Mar- 2006 | 14/1660 | 843·4 | 23/105 | 21·90 |
| **Endemic/ Wet, Serogroup W** |  |  |  |  |  |
| Leimkugel et al. 2007 | Nov-2003 | 0/140000 | 0·0 | 0/297 | 0·00 |
| Leimkugel et al. 2007 | Nov-2002 | 0/140000 | 0·0 | 0/319 | 0·00 |
| Leimkugel et al. 2007 | Nov-2005 | 0/140000 | 0·0 | 0/334 | 0·00 |
| Leimkugel et al. 2007 | Nov-2000 | 0/140000 | 0·0 | 0/301 | 0·00 |
| Leimkugel et al. 2007 | Nov-2001 | 0/140000 | 0·0 | 0/306 | 0·00 |
| Leimkugel et al. 2007 | Nov-2004 | 0/140000 | 0·0 | 2/313 | 0·64 |
|  |  |  |  |  |  |
| **Hyperendemic/ Dry, Serogroup W** |  |  |  |  |  |
| Boisier et al. 2006 | May-2003 | 5/7237 | 69·1 | 21/80 | 24·41 |
| Boisier et al. 2006 | Feb-2004 | 0/7469 | 0·0 | 7/70 | 10·00 |
| Hamidou et al. 2007 | Feb-2003 | 1/138057 | 0·7 | 13/287 | 4·53 |
| Hamidou et al. 2006 | Mai-2003 | 0/138057 | 0 | 13/272 | 4·78 |
| Hamidou et al. 2006 | Mar-2003 | 4/138057 | 2·9 | 8/277 | 2·89 |
| Leimkugel et al. 2007 | Apr-2004 | 0/140000 | 0·0 | 3/350 | 0·85 |
| Leimkugel et al. 2007 | Apr-2003 | 0/140000 | 0·0 | 0/312 | 0·00 |
| Leimkugel et al. 2007 | Apr-2000 | 0/140000 | 0·0 | 0/298 | 0·00 |
| Leimkugel et al. 2007 | Apr-2005 | 0/140000 | 0·0 | 0/321 | 0·00 |
| Leimkugel et al. 2007 | Apr-1999 | 0/140000 | 0·0 | 0/292 | 0·00 |
| Leimkugel et al. 2007 | Apr-1998 | 0/140000 | 0·0 | 1/301 | 0·33 |
| Leimkugel et al. 2007 | Apr-2001 | 0/140000 | 0·0 | 0/310 | 0·00 |
| Leimkugel et al. 2007 | Apr-2002 | 0/140000 | 0·0 | 0/339 | 0·00 |
| Mueller et al. 2006 | Mar-2003 | 7/253605 | 2·8 | 4/482 | 0·83 |
| Mueller et al. 2011 | Mar-2006 | 0/1660 | 0·0 | 0/105 | 0·00 |
| Mueller et al. 2011 | Mar-2006 | 0/4640 | 0·0 | 0/316 | 0·00 |
| Mueller et al. 2006 | Apr-2003 | 5/253605 | 2·0 | 6/469 | 1·28 |
| Mueller et al. 2006 | Feb-2003 | 4/253605 | 1·6 | 8/448 | 1·78 |
| Mueller et al. 2011 | Mar-2006 | 0/2600 | 0·0 | 0/203 | 0·00 |
| Sie et al. 2008 | Apr-2006 | 0/76847 | 0·0 | 0/316 | 0·00 |
| Trotter Trotter et al. 2013 | Feb 28-Mar 7 2008 | 0/623303 | 0·0 | 2/538 | 0·37 |
|  |  |  |  |  |  |
|  |  |  |  |  |  |
| **Authors. Publication Year [Reference]** | **Study**  **Month-Year** | **Monthly incidence** | | **Carriage prevalence** | |
|  |  | **cases/N** | **incid/100,000 pop** | **carriers/n** | **(%)** |
| **Endemic/ Wet, Serogroup X** |  |  |  |  |  |
| Leimkugel et al. 2007 | Nov-03 | 0/140000 | 0·0 | 3/297 | 1·01 |
| Leimkugel et al. 2007 | Nov-02 | 0/140000 | 0·0 | 2/319 | 0.63 |
| Leimkugel et al. 2007 | Nov-05 | 0/140000 | 0·0 | 0/334 | 0·00 |
| Leimkugel et al. 2007 | Nov-00 | 0/140000 | 0·0 | 33/301 | 10·96 |
| Leimkugel et al. 2007 | Nov-01 | 0/140000 | 0·0 | 4/306 | 1·31 |
| Leimkugel et al. 2007 | Nov-04 | 0/140000 | 0·0 | 0/313 | 0·00 |
|  |  |  |  |  |  |
| **Hyperendemic/ Dry, Serogroup X** |  |  |  |  |  |
| Boisier et al. 2006 | May-2003 | 0/7237 | 0·0 | 0/80 | 0·00 |
| Boisier et al. 2006 | Feb-04 | 0/7469 | 0·0 | 2/70 | 2·85 |
| Leimkugel et al. 2007 | Apr-2004 | 0/140000 | 0·0 | 0/350 | 0·00 |
| Leimkugel et al. 2007 | Apr-2003 | 0/140000 | 0·0 | 0/312 | 0·00 |
| Leimkugel et al. 2007 | Apr-2000 | 2/140000 | 1·4 | 52/298 | 17·45 |
| Leimkugel et al. 2007 | Apr-2005 | 0/140000 | 0·0 | 0/321 | 0·00 |
| Leimkugel et al. 2007 | Apr-1999 | 1/140000 | 0·7 | 10/292 | 3·42 |
| Leimkugel et al. 2007 | Apr-1998 | 0/140000 | 0·0 | 0/301 | 0·00 |
| Leimkugel et al. 2007 | Apr-2001 | 0/140000 | 0·0 | 49/310 | 15·80 |
| Leimkugel et al. 2007 | Apr-2002 | 0/140000 | 0·0 | 2/339 | 0·59 |
| Mueller et al. 2006 | Mar-2003 | 0/253605 | 0·0 | 1/482 | 0·21 |
| Mueller et al. 2011 | Mar-2006 | 0/1660 | 0·0 | 0/105 | 0·00 |
| Mueller et al. 2011 | Mar-2006 | 0/4640 | 0·0 | 0/316 | 0·00 |
| Mueller et al. 2006 | Apr-2003 | 0/253605 | 0·0 | 2/469 | 0·43 |
| Mueller et al. 2006 | Feb-2003 | 0/253605 | 0·0 | 0/448 | 0·00 |
| Mueller et al. 2011 | Mar-2006 | 0/2600 | 0·0 | 0/203 | 0·00 |
| Sié et al. 2008 | Apr-2006 | 0/76847 | 0·0 | 0/316 | 0·00 |
| Trotter et al. 2013 | Feb 28-Mar 7th 2008 | 0/623303 | 0·0 | 1/538 | 0·18 |
|  |  |  |  |  |  |
