# Supplementary material for: Incidence, Carriage and Case-Carrier Ratios for Meningococcal Meningitis in the African Meningitis Belt: A Systematic Review and Meta-Analysis
Source: PLoS One. 2015 Feb 6;10(2):e0116725. doi: 10.1371/journal.pone.0116725 (PMC4319942; doi:10.1371/journal.pone.0116725)

Protocol for Systematic Review
Version 1.0
May 2012

Case-carrier ratio for meningococci in the African meningitis belt – a systematic review and standardized data analysis

Lead reviewer
Thibaut Koutangni, Intern (MPH 2012);, EHESP School of Public Health, Department of Epidemiology and Biostatistics, Hôtel-Dieu, 1 place du Parvis Notre-Dame, 75181 Paris.
e-mail: thibautkoutangni@gmail.com

Study concept and design, epidemiologic and meningitis belt expertise, literature review
Judith Mueller, MD, MPH, PhD; Professeur en épidémiologie, EHESP School of Public Health, Department of Epidemiology and Biostatistics, Hôtel-Dieu, 1 place du Parvis Notre-Dame, 75181 Paris.
e-mail: judith.mueller@ehesp.fr

Advanced statistical methods and expertise for data synthesis.
Statistician/biostatistician with experience in meta-analysis

Active contributors:
Active contributors are researchers on study sites with relevant data who will provide additional information to the data collection.

Table of contents

Introduction: 4

1. Background and justification of this review 4

2. Objectives of the Review. 5

a. Main objective 5

b. Specific Objectives: 5

3. Methods. 5

3.1. Systematic literature review 5

3.1.1. Criteria for inclusion and exclusion of studies in the review. 5

3.1.2. Search Strategy 6

3.1.3. Electronic Search Strategy 7

3.1.4. Search Terms for Electronic Databases. 7

3.1.5. Study selection procedure: 7

3.1.6. Assessment of Methodological Quality of studies: 8

3.1.7. Data extraction 8

3.2. Contacting research groups for complementary data collection. 8

3.3. Compilation of the review database 8

3.4. Synthesis of the literature review and statistical analysis of database. 9

3.4.1. Quantitative synthesis of extracted data 9

3.4.1.1. Summary measures 9

3.4.1.2. Recalculation of point estimates and associated confidence intervals. 9

3.4.1.3. Estimation of cases-carriers ratios (CCR) 10

3.4.2. Meta-analysis of estimated case-carrier ratios 10

3.4.2.1. Assessment of heterogeneity across CCOU estimates. 10

3.4.2.2. Assessment of variability of the case carrier ratio estimates explained by study level characteristics (moderators). 10

3.4.2.3. Assessment of magnitude of variations of combined case-carrier ratios between epidemic situation and season. 11

3.5. Expanding analyses to advanced statistical methods. 11

3.6. Preparation of the manuscript for publication in an international peer-reviewed journal. 11

4. Project time frame 12

5. Plans for Updating the Review 12

6. Ethical considerations: 12

7. Partnership 12

8. Roles and responsibilities: 13

9. Data management: 13

10. Rules for publication and communication of data 13

11. Budget and funding source: 13
12. Conflict of interests 13
13. References 13
Figure 1: Flow diagram of study selection procedure. 15

Introduction:

1. Background and justification of this review

The high burden of meningococcal meningitis in the meningitis belt is related to a particular epidemiology of the disease[1]. It consists of high incidence during the dry season (seasonal hyperendemicity), localized epidemics and epidemic waves. Epidemics nearly always occur at the height of the dry with season with high temperatures and air dust load, and subside during the rainy season. They occur in form of localized epidemics in individual health center area or epidemics waves which affect entire regions at intervals of 7 to 10 years. Epidemics may occur in the same or adjacent area during the following dry season[2 3]. Due to its association with the dry season, climatic factors (including dust load and air humidity) are discussed as primary factors for hyperendemicity, while factors leading to epidemics remain hypothetic[4].

In a recent conceptual model[4], we suggested that the systematic transition from endemic incidence during the rainy season towards hyperendemic incidence during the dry season is due to increased risk of invasion given asymptomatic carriage. This asymptomatic infection of the nasopharynx, lasting for hours or months, is supposed to be a quasi-obligatory step toward invasive disease. Furthermore, the transition from hyperendemic incidence towards epidemic incidence during the dry season should be due to an increase in the carriage prevalence or acquisition rate in the population. In consequence, factors causing epidemics would have their impact primarily via the individual risk to acquire carriage, not via the host susceptibility to invasive disease given carriage. The evaluation of this hypothesis is crucial as control strategies of meningococcal meningitis, including vaccination strategies, could be designed or better targeted based on this model.

A methodological approach to evaluate the variation of strain invasiveness and host susceptibility and their relation with the epidemiology of the disease is the estimation of the case-carrier ratio across multiple populations depending on season and epidemic situation. The case-carrier ratio is considered an ecological proxy for the risk of disease given asymptomatic carriage of the causal infectious agent. It is estimated by combining disease incidence estimates with carriage prevalence estimates and therefore requires data from studies evaluating both simultaneously. Several research groups have conducted such studies during recent years, however, to date, no combined standardized analysis has been performed.

This review will therefore aim to retrieve and compile in a systematic way all relevant data from studies on meningococcal carriage and meningococcal meningitis surveillance that can be used to calculate case-carrier ratios for the African meningitis belt.

2. Objectives of the Review.

a. Main objective

To provide best evidence on how meningococcal case-carrier ratio varies according to season and epidemiologic situation in the African meningitis belt.

b. Specific objectives

- To identify existing serogroup-specific data on meningococcal carriage and concomitant meningococcal meningitis incidence within the meningitis belt through a systematic review.
- To estimate summary case-carrier ratios specific for epidemiologic situations (endemic, hyperendemic, epidemic) and seasons (dry and wet).
- To quantify variations of the case-carrier ratio between epidemiologic situations and seasons and relate the magnitude of variations to the epidemiology of meningococcal meningitis.

3. Methods.

This project holds the following work packages:

a. Conduct a systematic literature review to identify research groups with potentially eligible data and to extract published data.
b. Contact research groups for supplementary data collection.
c. Compile a data base
d. Analyze the data base by standardized descriptive analysis and meta-analysis) Expand analyses to more advanced statistical methods if appropriate.
e. Prepare an internship report and a manuscript for publication in an international peer-reviewed journal

3.1. Systematic literature review

3.1.1. Criteria for inclusion and exclusion of studies in the review.

Studies to be included in the review must contain the following elements:

Inclusion Criteria

Outcomes of interest: Studies will be eligible for inclusion if they report both pharyngeal meningococcal carriage and meningococcal meningitis incidence specifically by serogroups.

Time and place: We are interested in reviewing studies conducted in any country of the African meningitis belt as defined by Lapeyssonnie in 1963[5] and updated by A. Molesworth et al in 2002 [6]. Studies published from 1963 onward will be included in this review. This is because it is well recognized that the epidemiology of meningitis in Africa has been described for the first time by the French medical epidemiologist

Lapeyssonnie in 1963 after a long trip across the continent. Also the distinction between *N.meningitidis* and *N. lactamica* was not made until 1969. The Gambia is included in this review, although it may not really be part of the meningitis belt.

Study Participants: Study participants must belong to the general population eg: population of a village, city, district… and participants age must be defined (that is, age range of those from whom samples were taken were specified or studies took place in population groups of a known age, for example, high-school children, primary school children…)

Studies targeting children and/or young adults attending schools are also eligible for inclusion provided that the school attendance in this age group or population is generalised to allow extrapolation of study results to the general population in the age group.

Study Design. Studies with cross-sectional and longitudinal studies are eligible for inclusion.

Exclusion Criteria

- Studies not documenting both meningococcal meningitis incidence and carriage.
- Studies documenting meningococcal carriage but not meningitis incidence and for which attempts to obtain incidence data (same targeted population and time) has failed.
- Studies conducted outside the African meningitis belt (except the Gambia included for this exercise)
- Studies including only contacts of meningitis cases in the carriage evaluation.
- Studies targeting specific population groups such as prisoners, military camps etc…
- Studies with full text in language other than English and French

3.1.2. Search Strategy

Research publications, local and central government's and institution's publications and the grey literature are all targeted. No language restrictions will be placed on this search. Study identification will include both manual and electronic searching strategies. Electronic searches will involve the electronic databases and search terms listed below. Electronic search equation will be developed in such a way to be highly sensitive at first. For example keywords referring to study design of interest will not be included in search equations at first as some authors might not clearly state the cross-sectional design of their study in the abstract.

Any articles that are unsuitable can be excluded in the early stages or the search (for example, on the basis of abstracts and titles presented in electronic databases), whilst the decision to exclude or include other articles will only be made once the full article has been read. The number of articles included and excluded at the various stages as well as reasons for exclusion will be documented. Reference lists of publications will be hand-searched for studies and reviews.

3.1.3. Electronic Search Strategy

MEDLINE® with Full Text and Academic Search™ Complete (From 1963 to 2012) will be searched via EBSCOhost research platform. The African Index Medicus (AIM) an international Regional data base to African health literature and information sources will also be searched (from Start date to 2012). Google scholar (a non-bibliographic database) will be searched to identify relevant but not published literature including conference position papers, and government publications.

MEDLINE® with Full Text and Academic Search™ Complete will be searched for papers reporting confirmed cases of meningococcal meningitis and carriage of Neisseria meningitidis in defined age groups by the combination of medical subject headings (MeSH) terms, text words and synonyms terms. Three sets of (MeSH) terms and text words will be defined: 1) terms and text words to search for the first outcome of interest (meningococcal meningitis); 2) terms and text words to search for the second outcome of interest (nasopharyngeal carriage) and 3) terms to search for the geographic location of interest (countries of the meningitis belt).

First, the three sets of terms will be combined using Boolean operators to generate search equations either to broaden or to reduce the scope of the search. Second, generated search equations will be combined to get the global search equation for retrieving the maximum possible of relevant paper in electronic databases. The standard Boolean operators will be used to generate search equations.

A 'search diary' will be maintained detailing the names of the databases searched, the Keywords used, the search equation and results. Titles and abstracts of studies to be considered for retrieval will be recorded on **Mendeley desktop** along with details of references. (Mendeley desktop is a reference management application).

3.1.4. Search Terms for Electronic Databases.

The following terms (with wildcards when necessary) will be used when devising search strategies for electronic databases. The exact search terms and their results will be recorded as the search strategy is refined**. The basic search terms will be:**

(mening or mening* meningitis or cerebrospinal meningitis or Neisseria meningitis or acute meningitis or bacterial meningitis or epidemic mening*) and (mening* carri* or asymptomatic carri* or coloni?ation or neisseria colonisation carri* prevalence or pharyngeal coloni?ation or asymptomatic infection* or subclinical infection*) and (Africa or African meningitis belt or meningitis belt or Africa south of the Sahara or sub-Saharan Africa or Burkina Faso or Upper Volta or Niger or Mali or Togo or Ghana or Côte d'Ivoire or Ivory Coast or Senegal or Chad or Ethiopia or Sudan or Benin or Nigeria or Cameroun or The Gambia or Gambia).*

These basic search terms will be refined and updated as databases searches evolves and we discover new wording of the same key terms.

3.1.5. Study selection procedure.

The steps for study selection are provided below in **figure 1.** Studies will be selected for retrieval after abstracts and titles identified in electronic searches have been screened by

the lead reviewer for relevance. At this stage abstracts and titles that clearly have nothing to do with meningococcal meningitis will be excluded by the lead reviewer only. Studies that do make a reference to meningococcal meningitis will be considered for the literature review. Inclusion criteria will then be applied to those studies and study relevance for inclusion in quantitative data synthesis will be assessed independently by two reviewers.

3.1.6. Assessment of Methodological Quality of studies.

Coding and critical appraisal of studies to be included will be conducted by two reviewers using criteria agreed between them based on a review of methodological literature from the fields of epidemiologic surveys design and meningitis surveillance and formalized by a critical appraisal form. A codebook describing coding scales with example will be developed for study coding. If required, additional methodological information will be requested from studies' authors (eg: if the authors "Accounting for survey design in estimations of carriage prevalence"). When reviewers' conclusions over the validity of a study differ, the study will be reviewed jointly.

3.1.7. Data extraction

The lead reviewer will extract incidence and carriage prevalence data from articles eligible for inclusion in quantitative synthesis onto a pre-made excel sheet. Any further calculations on extracted study data considered necessary will be conducted by the lead reviewer and checked by the other reviewer.

3.2. Contacting research groups for complementary data collection.

Authors of relevant studies will be contacted in order to obtain either raw data or additional data needed. Research groups dealing with subjects related to meningitis in Africa will be contacted if possible to identify any work in progress or unpublished work or data on the review topic. This review will also be presented as a "work in progress" at relevant workshops or conferences (if possible) to help make researchers working in the review topic area, aware that the review is taking place.

3.3. Compilation of the review database

The database will be created in Excel. Statistical units will be "Cases and Carriers Observation Units" (CCOU) reported in publication/studies. For example, a publication reporting carriage prevalence and incidence estimates based on only one study protocol for 3 different carriage sampling time points will contribute 3 CCOU which are assigned to the same study.

For each CCOU, information will be recorded for the following variables in the database:

- Study/publication code (assigned by reviewers)
- Source (main author and year of the study/publication reporting the CCOU)
- Participants (general population/school children…)
- Age range of participants in years)
- Follow up/sampling time point of the carriage study
- Inclusion criteria for the carriage study
- Carriage study sample size

- Size of the population covered by meningitis surveillance
- Setting of the CCOU
- Epidemiologic context (endemic, hyper endemic , epidemic or post epidemic)
- Local season corresponding to the CCOU
- *Nisseria meningitidis* (*N.m*) serogroup-specific carriage prevalence
- *N.m* Serogroup-specific cumulative incidence during the month of the carriage study
- Serogroup-specific carriage prevalence by defined age groups ([0-4], [5-19], and [20+ years)
- *N.m* serogroup-specific Cumulative incidence during the month of the carriage study by defined age groups ([0-4], [5-19], and [20+ years).

We will also record for each CCOU, other information that could confound carriage, incidence and case-carrier ratios estimates, these will include:

- Year (year toward which CCOU data reports)
- Mass campaign vaccination in the target population of the CCOU during the three last years (yes/No/unknown; and years since last campaign (1-2-3))
- Type of vaccine used (A/C polysaccharide A/C , A conjugate vaccine …)
- Estimated vaccination coverage in percentage
- Time since first rain before carriage study onset

3.4. Synthesis of the literature review and statistical analysis of database.

Summary characteristics of CCOUs included in this review will be presented in a table, along with quality assessment. Carriage prevalence and incidence estimates and case-carrier ratios with 95% confidence intervals will be plotted using appropriate graphs. Estimated summary case-carrier ratios with 95% confidence intervals will be plotted.

Synthesis of result will be in form of narrative synthesis and quantitative data synthesis. For quantitative data synthesis, we will use a standardized descriptive data analysis as eligible studies for quantitative synthesis likely will be heterogeneous.

If appropriate, we will add a meta-analysis approach to summarize quantitative data from the review. and compare conclusions from both approaches.

3.4.1. Quantitative synthesis of extracted data

3.4.1.1. Summary measures

The ratio of clinical cases of meningococcal meningitis to asymptomatic carriers (cases-carriers ratio) is the primary estimate of interest in this review. However such ratio will not be directly reported in potentially eligible studies but can be computed from point estimates or data (cumulative incidence and prevalence) reported in eligible studies.

3.4.1.2. Recalculation of point estimates and associated confidence intervals.

For each eligible CCOU, serogroup-specific cumulative incidences will be reported as number of clinical confirmed cases per thousand population. While most CCOU will report serogroup-specific carriage prevalence in defined age groups, some will not. In such

situations and when a sampling design other than a simple random sampling method is performed, authors will be contacted in order to get serogroup-specific prevalence estimates with 95% confidence interval by defined age groups accounting for their sampling design. For simple random design reviewers will estimate the 95% confidence interval for point estimates If not reported by authors. Reported carriage prevalence is usually in percentage (%). But in this review it will be recalculated and reported as the number of asymptomatic carriers per thousand population (‰). This is to standardize the numerator and the denominator of the case-carrier ratio to the same population size (1000) in order to facilitate data synthesis and interpretation. Both estimates (cumulative incidence and prevalence) will be standardized according to three age strata (< 5 ; [5-19] and 20+year-old) when information is available for the CCOU in the publication or provided by author after a request.

3.4.1.3. Estimation of cases-carriers ratios (CCR)

For each CCOU, the cases-carriers ratio and associated 95% confidence interval will be estimated.

3.4.2. Meta-analysis of estimated case-carrier ratios

We will use a fixed effect model for reasons of statistical simplicity in comparison to a random effect model. A weighted average of cases-carriers ratios will be estimated according to meningitis season and epidemic situation. Weighting variable for each cases-carrier ratio will be the carriage survey sample size. If all selected studies reports standard errors of carriage estimates, then the inverse of the variance will be used as weighting variable instead of the carriage survey sample size. The 95% CI of the weighted average case-carrier ratio will be estimated as well. The weighted average will be compare to the unweighted mean to see if there is a significant difference.

3.4.2.1. Assessment of heterogeneity across CCOU estimates.

We will use the heterogeneity statistic Q to test the hypothesis that there is a common population case-carrier ratio estimate across CCOU according to epidemiologic situations and season. Heterogeneity between case-carrier ratio estimates will also be assessed visually by looking at how much their associated 95% confidence intervals overlap. Poor overlap or outliers may indicate statistical heterogeneity. Inconsistency will be quantified using the I^2 statistic.
If there is substantial heterogeneity (50% to 90 %) we will conduct subgroup analysis and compare the combined estimates between subgroups. Subgroups will be determined by study characteristics assessed during coding and critical appraisal of selected studies. In case of little or moderate heterogeneity (<50 %) the non-weighted mean of case-carrier ratios will be consider.

3.4.2.2. Assessment of variability of the case carrier ratio estimates explained by study level characteristics (moderators).

Study characteristics assessed during studies coding as well as characteristics known to potentially affect the case-carrier ratio point estimates will be tested. The outcome variable will be the case-carrier ratio estimated from each study and the independent variables will be study characteristics/moderators. For categorical study characteristics (assessed during study coding) an ANOVA test will be used (we assume here that

statistical conditions are meet). For continuous study characteristics variables a correlation test will be used to assess a possible linear relation with case carrier-ratio point estimates.

3.4.2.3. Assessment of magnitude of variations of combined case-carrier ratios between epidemic situation and season.

The ratio of combined or summary cases-carrier ratios between epidemic situation and seasons will provide an approximation of the increase in the risk of meningitis given colonization by a hyper virulent strain between epidemic situation and seasons in the African meningitis belt.

3.5. Expanding analyses to advanced statistical methods.

Decision for the use of advanced statistical model or methods for data analysis will be discussed by reviewers.

3.6. Preparation of the manuscript for publication in an international peer-reviewed journal.

The lead reviewer will prepare a first draft of the manuscript. The first draft will be proof read by co-authors and necessary corrections will be made. Co-authors must agree on the content of final manuscript before its submission for publication. Active contributors who have contributed to interpretation and manuscript preparation will be included as co-authors (on positions other than first, second or last author).

The manuscript will be sent for information to all other active contributors one week before submission.

A master thesis report will be prepared by the lead reviewer, proof read by the co-reviewer and submitted for master thesis defense at the Ecole des Hautes Etudes de Santé Publique (EHESP) by June 11 , 2012. For the thesis report only published CCOU for which complementary data needed are obtained prior to June will be included in quantitative analysis.

4. Project time frame

The review is expected to take 6 months to complete.

Tasks to complete	Février	Mars	Avril	Mai	Juin	July
Litterature review	←	—	—	—	→	
Data Extraction from litterature		←→				
Additional data collection from authors		←	—	→		
Data base compilation , and statistical analysis			←→			
Thesis writing		←	—	—	→	
Thesis correction by thesis supervisor				←	→	
Thesis submission to EHESP					Before June 11th	
Thesis defense at EHESP						Early July
Preparation of manuscript for publication						September through December 2012

5. Plans for Updating the Review

This review will be updated until submission of a manuscript.

6. Ethical considerations:

This review aims at using aggregated published data but also data containing personal information of studies participants such as age of participants (if raw data where obtained from partners or publication authors). Therefore, the review team is committed to accept only fully anonymized databases, to keep all these data confidential and publish only aggregated data or results from statistical analysis.

7. Partnership

The research project will take place in an informal collaboration with interested researchers from different institutions, Eligible data are known to exist from at least three study sites in the African meningitis belt (Centre Muraz/AMP in Burkina Faso, Navrongo/STI in Ghana, CERMES/RII Pasteur in Niger) and sub-Saharan Africa (MRC in The Gambia). Data sharing agreements with these research groups will be formulated to obtain raw or aggregated original data.

8. Roles and responsibilities:

Thibaut Koutangni will be in charge of conducting the systematic literature review, extracting data, designing additional data collection tools, compiling the review database, conducting standardized data analysis and metaanalysis, writing data sharing agreement, and preparing the first draft of the manuscript.

Judith Mueller will supervise the project, provide epidemiological expertise, assist with contacting research groups and proof read the manuscript.

The statistician who will be part of the review team will provide guidance on methods of data synthesis and advanced statistical methods, and proof read the manuscript as well. Active contributors are invited to co-authorship by providing relevant expertise for data interpretation and review the first draft of the manuscript.

9. Data management:

The database created in the framework of this project as well as data obtained from active contributors will be accessible only to the two reviewers.

The study data base will be property of the EHESP, but any use outside the scope of data sharing agreements will require agreement from contributing research sites.

10. Rules for publication and communication of data

Will be listed as authors of the manuscript only participants in the review process, synthesis of data and interpretation of results. The final manuscript will be send to contributors and partners before publication. If they wish, partners could designate a person who will participate in data synthesis, interpretation of results and contribute in the manuscript. This will lead to inclusion of the name of the designated person in the author's list (place to be define except first and last place)

11. Budget and funding source:

This project has not received any specific grant. It is being conducted in the framework of a Master of Public Health practicum, and the lead reviewer will receive a monthly gratification from EHESP.

12. Conflict of interests

Reviewers are unaware of any potential conflict of interests.

13. References

1 Greenwood B. Manson Lecture : Meningococcal meningitis in Africa. *Transactions of the Royal Society of Tropical Medicine and Hygiene* 1999;**93**:341-53.

2 Greenwood B, Bradley A, and Wall A. Meningococcal disease and season in sub-Saharan Africa. *The Lancet* 1985;**ii**:829-830.

3 Jackou-Boulama M, Michel R, Ollivier L, Meynard J. B, Nicolas P, Boutin J. P. Correlation between rainfall and meningococcal meningitis in Niger. Médecine tropicale revue du Corps de santé colonial 2005;**65**:329-33.

4 Mueller JE, Gessner BD. A hypothetical explanatory model for meningococcal meningitis in the African meningitis belt. *International Journal of Infectious Diseases* 2010;**14:**553-9.

5 Lapeyssonie L. La méningite cérébro-spinale en afrique. *Bull World Health Organization* 1963;**28**:3-144.

6 Molesworth AM et al. Where is the meningitis belt? defining an area at risk of epidemic meningitis in Africa. *Transactions of the Royal Society of Tropical Medicine and Hygiene* 2002;**96**:42-249.

Figure 1: Flow diagram of study selection procedure.

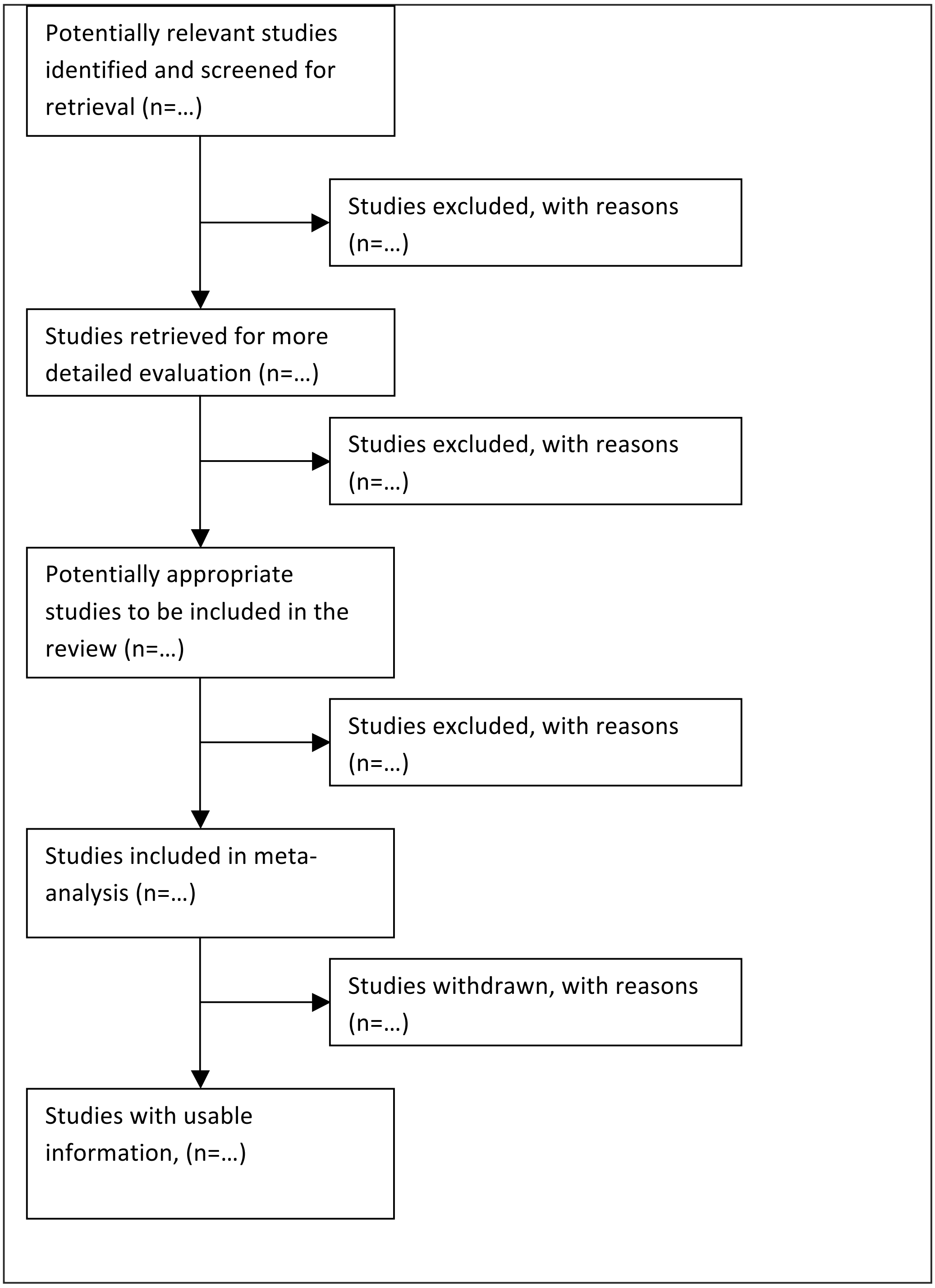

Supplement: S1 Text — (PDF) [file pone.0116725.s006.pdf]
