# Supplementary material for: Incidence, Carriage and Case-Carrier Ratios for Meningococcal Meningitis in the African Meningitis Belt: A Systematic Review and Meta-Analysis
Source: PLoS One. 2015 Feb 6;10(2):e0116725. doi: 10.1371/journal.pone.0116725 (PMC4319942; doi:10.1371/journal.pone.0116725)
Supplement: S2 Text — (DOCX) [file pone.0116725.s007.docx]

**Text S2. Search strategy**

**Medline via EBSCOhost research platform**

**#1)** SH Meningitis, Meningococcal

**#2)** TI (Meningitis, Meningococcal, Serogroup Y) or TI (Serogroup Y, Meningococcal Meningitis) or TI (Meningococcal Meningitis, Serogroup Y) or TI (Meningitis, Meningococcal, Serogroup C) or TI (Serogroup C Meningococcal Meningitis) or TI (Meningococcal Meningitis, Serogroup C)

**#3)** TI (Meningitis, Meningococcal, Serogroup B) or TI (Serogroup B Meningococcal Meningitis) or TI (Meningococcal Meningitis, Serogroup B)

**#4)** TI (Meningitis, Meningococcal, Serogroup A) or TI (Serogroup A Meningococcal Meningitis) or TI (Meningococcal Meningitis, Serogroup A)

**#5)** TI (Meningococcal Meningitis, Serogroup W 135) or TI (Serogroup W-135, Meningococcal Meningitis) or TI (Serogroup W 135)

**#6)** TI (Meningitis, Meningococcal, Serogroup X) or TI (Serogroup X Meningococcal Meningitis) or TI (Meningococcal Meningitis, Serogroup X)

**#7)** (#2 or #3 or #4 or #5 or #6)

**#8)** AB (Meningitis, Meningococcal, Serogroup X) or AB (Serogroup X Meningococcal Meningitis) or AB (Meningococcal Meningitis, Serogroup X)

**#9)** AB (Meningitis, Meningococcal) or AB (Meningococcal Meningitis) or AB (*Neisseria meningitis*) or AB (Meningitis, Cerebrospinal) or AB (Acute meningitis) or AB (Epidemic meningitis) or AB (Meningitis, Meningococcic)

**#10)** TI (Meningitis, Meningococcal) or TI (Meningococcal Meningitis) or TI (*Neisseria meningitis*) or TI (Meningitis, Cerebrospinal) or TI (Acute meningitis) or TI (Epidemic meningitis) or TI (Meningitis, Meningococcic)

**#11)** AB (Meningitis, Meningococcal, Serogroup Y) or AB (Serogroup Y, Meningococcal Meningitis) or AB (Meningococcal Meningitis, Serogroup Y) or AB (Meningitis, Meningococcal, Serogroup C) or AB (Serogroup C Meningococcal Meningitis) or AB (Meningococcal Meningitis, Serogroup C)

**#12)** AB (Meningococcal Meningitis, Serogroup W 135) or AB (Serogroup W-135, Meningococcal Meningitis) or AB (Serogroup W 135) or

**#13)** AB (Meningitis, Meningococcal, Serogroup A) or AB (Serogroup A Meningococcal Meningitis) or AB (Meningococcal Meningitis, Serogroup A)

**#14)** AB (Meningitis, Meningococcal, Serogroup B) or AB (Serogroup B Meningococcal Meningitis) or AB (Meningococcal Meningitis, Serogroup B)

**#15)** (#8 or #9 or #10 or #11 or #12 or #13 or #14)

**#16)** #7 or #15

**#17)** #1 and #16

**#18)** MH Africa/ or MH African meningitis belt/ or MH meningitis belt/ or MH Africa south of the Sahara/ or MH sub-Saharan Africa / or MH Burkina Faso/ or MH Niger/ or Niamey/ or MH Mali/ or MH Togo/ or MH Ghana/ or MH Côte d’Ivoire/ or MH Ivory Coast/ or MH Senegal/ or MH Chad/ or MH Ethiopia/ or MH Sudan/ or MH Benin/ or MH Nigeria/ or MH Cameroun/ or MH The Gambia/ or MH Gambia/

**#19)** #17 and #18

**Academic Search complete via EBSCOhost research platform**

**#1)** DE "Meningitis, Cerebrospinal"

**#2)** TI (Meningitis, Meningococcal, Serogroup Y) or TI (Serogroup Y, Meningococcal Meningitis) or TI (Meningococcal Meningitis, Serogroup Y) or TI (Meningitis, Meningococcal, Serogroup C) or TI (Serogroup C Meningococcal Meningitis) or TI (Meningococcal Meningitis, Serogroup C)

**#3)** TI (Meningitis, Meningococcal, Serogroup B) or TI (Serogroup B Meningococcal Meningitis) or TI (Meningococcal Meningitis, Serogroup B)

**#4)** TI (Meningitis, Meningococcal, Serogroup A) or TI (Serogroup A Meningococcal Meningitis) or TI (Meningococcal Meningitis, Serogroup A)

**#5)** TI (Meningococcal Meningitis, Serogroup W 135) or TI (Serogroup W-135, Meningococcal Meningitis) or TI (Serogroup W 135)

**#6)** TI (Meningitis, Meningococcal, Serogroup X) or TI (Serogroup X Meningococcal Meningitis) or TI (Meningococcal Meningitis, Serogroup X)

**#7)** (#2 or #3 or #4 or #5 or #6)

**#8)** AB (Meningitis, Meningococcal, Serogroup X) or AB (Serogroup X Meningococcal Meningitis) or AB (Meningococcal Meningitis, Serogroup X)

**#9)** AB (Meningitis, Meningococcal) or AB (Meningococcal Meningitis) or AB (*Neisseria meningitis*) or AB (Meningitis, Cerebrospinal) or AB (Acute meningitis) or AB (Epidemic meningitis) or AB (Meningitis, Meningococcic)

**#10)** TI (Meningitis, Meningococcal) or TI (Meningococcal Meningitis) or TI (*Neisseria meningitis*) or TI (Meningitis, Cerebrospinal) or TI (Acute meningitis) or TI (Epidemic meningitis) or TI (Meningitis, Meningococcic)

**#11)** AB (Meningitis, Meningococcal, Serogroup Y) or AB (Serogroup Y, Meningococcal Meningitis) or AB (Meningococcal Meningitis, Serogroup Y) or AB (Meningitis, Meningococcal, Serogroup C) or AB (Serogroup C Meningococcal Meningitis) or AB (Meningococcal Meningitis, Serogroup C)

**#12)** AB (Meningococcal Meningitis, Serogroup W 135) or AB (Serogroup W-135, Meningococcal Meningitis) or AB (Serogroup W 135) or

**#13)** AB (Meningitis, Meningococcal, Serogroup A) or AB (Serogroup A Meningococcal Meningitis) or AB (Meningococcal Meningitis, Serogroup A)

**#14)** AB (Meningitis, Meningococcal, Serogroup B) or AB (Serogroup B Meningococcal Meningitis) or AB (Meningococcal Meningitis, Serogroup B)

**#15)** (#8 or #9 or #10 or #11 or #12 or #13 or #14)

**#16)** #7 or #15

**#17)** #1 and #16

**#18)** ZG “Africa” or ZG “African meningitis belt” or “ZG meningitis belt” or ZG “Africa south of the Sahara” or ZG “sub-Saharan Africa” or ZG “Burkina Faso” or ZG “Niger” or ZG “Niamey” or ZG “Mali” or ZG “Togo” or ZG “Ghana” or ZG “Côte d’Ivoire” or ZG “Ivory Coast” or ZG “Senegal” ZG “Chad” or ZG “Ethiopia” or ZG “Sudan” or ZG “Benin” or ZG “Nigeria” or ZG “Cameroun” or ZG “The Gambia” or ZG “Gambia”

**#19)** #17 and #18

**African Medicus Index.**

Meningitis [Descriptor] or Meningite [Descriptor] or *Neisseria meningitidis* [Descriptor] or meningitis [Key Word] or meningite [Key Word] or *Neisseria meningitidis* [Key Word] or meningitis [Title] or meningococcal [Title] or meningococcic [Title] or méningite [Title] or *Neisseria* [Title] or *Neisseria* and meningitidis [Title]
